# Validation of a Mechanistic Model for Non-Invasive Study of Ecological Energetics in an Endangered Wading Bird with Counter-Current Heat Exchange in its Legs

**DOI:** 10.1371/journal.pone.0136677

**Published:** 2015-08-26

**Authors:** Megan J. Fitzpatrick, Paul D. Mathewson, Warren P. Porter

**Affiliations:** Department of Zoology, University of Wisconsin-Madison, Madison, Wisconsin, United States of America; Coastal Carolina University, UNITED STATES

## Abstract

Mechanistic models provide a powerful, minimally invasive tool for gaining a deeper understanding of the ecology of animals across geographic space and time. In this paper, we modified and validated the accuracy of the mechanistic model Niche Mapper for simulating heat exchanges of animals with counter-current heat exchange mechanisms in their legs and animals that wade in water. We then used Niche Mapper to explore the effects of wading and counter-current heat exchange on the energy expenditures of Whooping Cranes, a long-legged wading bird. We validated model accuracy against the energy expenditure of two captive Whooping Cranes measured using the doubly-labeled water method and time energy budgets. Energy expenditure values modeled by Niche Mapper were similar to values measured by the doubly-labeled water method and values estimated from time-energy budgets. Future studies will be able to use Niche Mapper as a non-invasive tool to explore energy-based limits to the fundamental niche of Whooping Cranes and apply this knowledge to management decisions. Basic questions about the importance of counter-current exchange and wading to animal physiological tolerances can also now be explored with the model.

## Introduction

Local environments play a critical role in animals’ growth, reproduction, activity patterns, food requirements, and location in space and time. However, a comprehensive view of mechanistic relationships between environments and animals is often lacking. A mechanistic (explaining phenomena only by reference to physical or biological causes) understanding of animal ecology, incorporating the effects of individual environmental, morphological, physiological, and behavioral factors on energy and mass balances, can deepen understanding of basic ecological questions. Further, a mechanistic view can inform conservation efforts for endangered and threatened species. A mechanistic approach to ecological energetics, for example, can help managers not only identify habitat requirements [1] but also better understand species’ responses to changing environmental conditions [1,2,3] and stressors [4].

Mechanistic models of local microclimates (i.e. models incorporating variation in conditions throughout the day and the varied conditions available to an animal in its habitat, such as varying shade levels or water depths) and animal heat and mass balances can be used to help obtain a comprehensive understanding of the factors governing relationships between animals and their environments. An advantage of such models is that they allow users to vary individual environmental, behavioral, and physiological factors. This allows for more thorough exploration of their effects on mass and energy balances and the subsequent behavioral, physiological, and ecological ramifications. These effects can be explored on coarse or fine time scales (i.e. years or hours) and across coarse or fine resolution geographic space. Importantly for endangered species with reduced ranges, simulations of novel environmental conditions (e.g. proposed reintroduction locations for endangered species or predicted climate change scenarios) may be more accurate than those of correlative models, which may need to be extrapolated beyond available data to simulate novel conditions [5]. In particular, a mechanistic understanding of a species’s fundamental niche may reveal potential range beyond that revealed by correlations based on current realized niche conditions. Mechanistic models also minimize disturbance to wild animals compared to common methods of measuring total energy expenditure, such as the doubly-labeled water technique and heart-rate telemetry. Following initial model parameterization, which can often be achieved with captive animals and study specimens (e.g. [6,7]), no animal handling is required.

In this study, we use the mechanistic modeling program Niche Mapper. Niche Mapper consists of two coupled submodels, a microclimate model and a model of animal heat and mass balances that includes behavioral, postural and physiological responses to diurnal environmental changes. The microclimate model has previously been used in combination with other process-driven models to explore the relative contributions of climate change and evolutionary effects on mosquito distributions [8] and to determine future suitable nest sites for tuataras [9]. Both models have been used to determine future suitable habitats for the Western Swamp Tortoise [10] and to define current and future distribution limits on land and at sea for leatherback sea turtles [11]. They have also been used to study polar bears’ survivability in the Arctic with global warming [7], the distribution of the endangered Serow deer in Japan [12], and the spatial behavior of elk in desert-like and montane regions [13].

In this study, we evaluate Niche Mapper’s ability to accurately simulate the energy expenditure of Whooping Cranes (*Grus americana*). Whooping Cranes are a large endangered species, native to North American wetlands, that is being managed via multiple reintroduction attempts. A mechanistic model that could be used to simulate crane energetics under varying environmental conditions and stressors would be useful in informing evidence-based management decisions. A model of energy expenditure in this once wide-ranging species would also be useful to evaluate basic ecological questions about the relationship between timing of spring migration, fat storage, and latitude of breeding grounds in birds.

For birds, Niche Mapper has previously been shown to simulate energy expenditure accurately compared to indirect calorimetry measurements of Hawaiian honeycreepers (*Hemignathus virens*, *H*. *parvus*) [6] and doubly-labeled water measurements of Great Cormorants (*Phalacrocorax carbo*) [14]. However, Whooping Cranes differ from these species in that they have particularly long legs. Long, unfeathered legs can be a significant source of heat loss compared to insulated portions of the body, and many birds use a “counter-current exchange system” of close-running arteries and veins to reduce heat loss from the legs [15,16,17,18]. Heat is lost from warm blood in arteries to the adjacent, cooler veins, allowing leg temperature to approach ambient temperature in lower portions of the leg, decreasing convective and radiant heat loss to the environment. This system may be particularly important for wading birds, as convective heat loss to water occurs much faster than heat loss to air at the same temperature and velocity [19]. For some species, heat loss to the environment from legs can be increased in warm ambient temperatures by shunting venous blood flow to superficial veins nearer the skin surface, and sometimes by shifting more arterial flow to non-counter-current arteries [18]. This shunting reduces counter-current heat exchange and allows the average leg temperature to warm, helping to prevent heat stress by increasing heat loss to the environment via unfeathered legs.

Niche Mapper has been used to simulate the energy expenditure of a large bird with long bare legs, the greater flamingo (*Phoenicopterus roseus*) [20]. However, flamingos were modeled during cold spells when water was frozen and birds were thus not wading. Niche Mapper has not previously been used to model large wading birds like the Whooping Crane.

Here we describe modifications to Niche Mapper to enable simulations of wading animals with counter-current heat exchange in their appendages. We then test the model through comparisons of model predictions to data collected from live Whooping Cranes. Specifically, we compare predicted energy expenditure to expenditure (i) measured using the doubly-labeled water technique and (ii) predicted based on time-energy budgets. We further compare model-simulated leg temperatures to leg temperatures observed using infrared photography. For reference, lower critical temperatures and minimal conduction values were simulated in metabolic chamber-like conditions (i.e. low wind speed, low relative humidity, no solar radiation) and compared to predictions based on previously published allometric equations for birds. We also simulated the energy expenditures of cranes in metabolic chamber-like conditions with and without counter-current heat exchange, and with and without wading in water of varying depths. These simulations were performed to find out how the new model additions affect simulated animal metabolic rates when all other parameters (particularly microclimate) were kept constant. This allowed us to better evaluate how the new model additions affected simulated metabolic rates of our study cranes. Finally, sensitivity analyses were also carried out to assess the importance of various input variables on simulated energy expenditures.

## Materials and Methods

All study protocols involving animal subjects were approved by the Animal Care and Use Committee of the University of Wisconsin-Madison (Protocol Number: L00450-0-07-12) and the International Crane Foundation Animal Care and Use Committee (Protocol Number: 2012–01).

### Doubly-Labeled Water Measurements

Doubly-labeled water measurements were obtained from two captive cranes (one adult male and one adult female) on display at the International Crane Foundation (Baraboo, WI) on September 24–28, 2012. This pair of cranes had access to a pond and frequently waded, allowing us to test Niche Mapper’s ability to model the energy expenditure of wading birds.

The doubly-labeled water method of measuring energy expenditure has been described previously by a number of authors (e.g. [21,22]). In this study, doses of approximately 0.10 g H_2_^18^O (97 AP) kg^-1^ and 0.13 g D_2_O (99.9 AP) kg^-1^ were given as a mixture. The mixture was diluted 5:2 with 3% physiological saline and delivered via intravenous injection to the jugular vein. Blood samples were taken from the jugular vein prior to doubly-labeled water dosing, approximately 2.5 hours after dosing, and four days after the first post-dose sample. The 4.1 day period between doubly-labeled water injection and the second blood sample corresponded to approximately two biological half-lives of each isotope. The daily rates of decline in deuterium, k_d_ [22], were 0.308δ for the male crane and 0.283δ for the female crane (S3 Table), corresponding to half-lives of 2.2 days and 2.4 days. The daily rates of decline in ^18^O, k_o_ [22], were 0.360 for the male crane and 0.333 for the female crane (S3 Table), corresponding to half-lives of 1.9 and 2.1 days. Thus, the sample period was just below two biological half-lives for deuterium in both cranes, just above two biological half-lives of ^18^O for the male crane, and just below two biological half-lives of ^18^O for the female crane.

Samples were analyzed using isotope ratio mass spectrometry. Samples were spun to separate the plasma and passed through a 10kDa exclusionary filter (Vivaspin GE Healthcare) to remove the large protein portion prior to analysis. Deuterium was measured using a Thermo Scientific DeltaPlus isotope ratio mass spectrometer and ^18^O was measured using a Thermo Scientific Delta V. CO_2_ expiration rates were calculated from changes in isotope concentration using the one-pool model Equation 7.17 in Speakman [22]. Calculations using a two-pool model resulted in average energy expenditures of 1.1–1.3 times basal metabolic rate. The one-pool model was judged to be a better model of Whooping Crane physiology given that 1.1–1.3 times basal metabolic rate is much lower than the energy expenditure values calculated from observed time-activity budgets for the cranes (see Results section) and the energy expenditures measured for captive Whooping Cranes in Nelson [23]. The size of each crane’s body water pool was determined using the intercept method [22].

To relate CO_2_ expiration rates to energy expenditure, the composition of the subjects’ diets during the experiment period must be known. During the experiment, the cranes were provided ad libitum with food pellets in a bucket located inside a shelter adjacent to their enclosure (as usual). Food buckets were weighed at the beginning of the experiment period and before and after keepers added food each day. Changes in food bucket weight were used to determine the total mass of pellets consumed by the pair. A sample of food kept in a room adjacent to the shelter was weighed near sunrise and during early afternoon (between 13:00 and 15:00) to monitor for changes in weight with daily humidity fluctuations. The food lost less than 1% mass during the first afternoon of the study and then did not change in mass for the rest of the study period, so no humidity-related adjustments were made to measured food bucket weights.

The cranes also foraged for food items in their enclosure. However, a respiratory quotient based on the composition of food pellets was used to interpret doubly-labeled water results because the composition of non-pellet food items made little difference in the final energy expenditure calculations (S1 Text). Energy content of substrates, oxygen and carbon dioxide utilized per gram of substrate, and respiratory quotients for all calculations were obtained from Schmidt-Nielson [24].

### Model Overview

Niche Mapper consists of two coupled submodels, including a model that simulates hourly microclimate conditions available to an animal and a biophysical/behavioral animal model that simulates heat and water exchanges between an animal and its microclimate, along with associated energy expenditure, food, and water requirements. We used the model version for endothermic animals. Niche Mapper was originally developed by Beckman *et al*. [25] and Porter *et al*. [26] based on early generic animal models of Porter and Gates [27], a generic solar radiation model by McCullough and Porter [28], and a boundary layer theory [29]. It has been modified by Porter and Mitchell [30].

The microclimate model simulates environmental conditions that will affect an animal’s heat (energy) and mass balances, based on user-input climate, topographic, vegetation, and soil parameters. We used version Micro2010a of the microclimate model, which was most recently modified by Fuentes and Porter [31]. The component equations of the microclimate model are described in Porter *et al*. [26], and the subroutines are described in Fuentes and Porter [31]. In brief, output microclimate variables are calculated at the animal’s location (height above or below ground) in the environment on an hourly basis for each day being modeled. Key output variables include hourly air temperature, relative humidity, wind speed, solar radiation, ground temperature, and sky temperature. Hourly air temperatures, wind speed, relative humidity, and cloud cover are calculated by fitting sinusoidal curves to user-input daily minimum and maximum values, given user-input times of minimum and maximum values relative to solar noon and sunrise for each day. Solar radiation is calculated based on latitude/longitude, Julian day, percent cloud cover, elevation, and slope and aspect of the ground, as described in McCullough and Porter [28]. Soil temperatures are calculated at a series of user-defined depths from the surface to deep soil, typically 2m.

The biophysical/behavioral model for endotherms is described in detail in Mathewson and Porter [7] and Mathewson [32]. In summary, the model uses hourly output from the microclimate model to calculate the metabolic rate necessary for the animal to maintain its user-input core temperature during each hour of each day being modeled. Heat exchanges with the environment via convection, conduction, solar radiation, longwave infrared radiation, and respiratory evaporation, and cutaneous evaporation are simulated. Within the feather (or fur) layer, heat transfer via simultaneous air and hair conduction and infrared radiation is simulated. A skin-to-core temperature gradient within the animal body is calculated by assuming distributed heat generation in the flesh from animal metabolism. Subcutaneous fat acts as an insulating, non-heat generating, layer between the inner flesh and skin surface.

**Fig 1:**
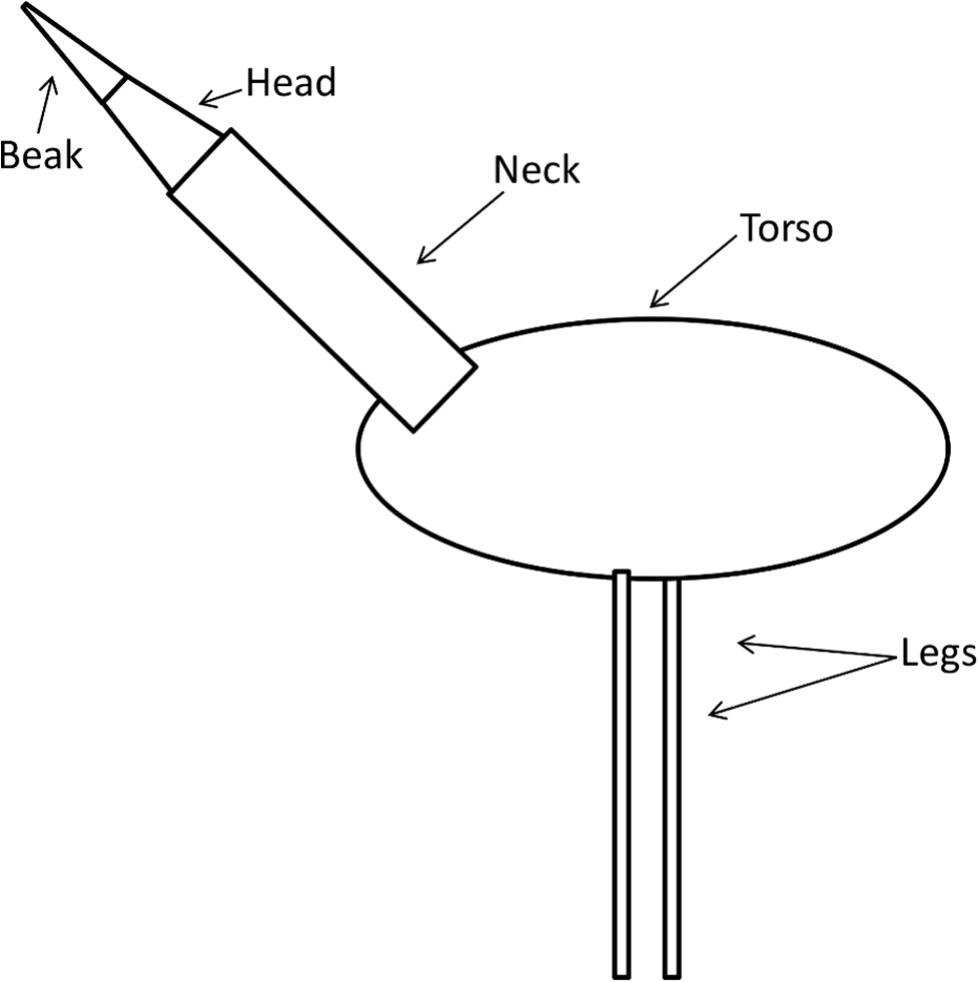
Diagram of the shapes used to model Whooping Crane body parts. Body parts are drawn in proportion to the dimensions of the female crane measured in this study.

Animals with multiple distinct body parts (e.g. torso, appendages, neck) like Whooping Cranes are modeled as a connected group of simple shapes with well known heat transfer properties (e.g. [33]). Whooping Cranes were modeled with an ellipsoidal torso, a cylindrical neck, cylindrical legs, and a cone-shaped head (Fig 1). We modeled the proximal part of the head as a feathered, truncated cone and the distal part of the head as a conical unfeathered beak. Whooping Cranes were also modeled with a thin layer of subcutaneous fat on the torso. Daily energy spent on activity (as a multiple of basal metabolic rate) was incorporated into the energy requirement and heat production calculations.

When the animal’s simulated hourly metabolic rate is greater than the minimum value required for basal functions and activity, iterative thermoregulatory mechanisms to conserve heat are engaged. Simulated Whooping Cranes were allowed to engage in ptiloerection, engage in vasoconstriction, and slightly decrease core temperature. Similarly, iterative thermoregulatory mechanisms to “cool” the animal (e.g. vasodilation) engage when the simulated metabolic rate and associated heat production cannot be maintained without an increase in core temperature. Simulated Whooping Cranes were allowed to engage in vasodilation, slightly increase body core temperature, and pant.

Final hourly energy expenditures are integrated across the 24 hours of a day to calculate daily values.

### Model Modifications for Wading and Counter-Current Exchange

Niche Mapper was updated to model appendages with counter-current exchange systems and to allow these appendages to wade in water.

In Niche Mapper, endotherm body parts are typically modeled with a constant core temperature equal to deep body temperature. In real animals, a counter-current heat exchange system leads to a temperature gradient down the length of an appendage. In effect, this causes average appendage temperature to decrease compared to the average temperature of other body parts [34]. This reduces the difference between skin and air (or water) temperature. To simulate effects of counter-current heat exchange, we modified Niche Mapper to allow the core temperature of appendages to drop to a user-input value above local ambient temperature, simulating a decreased average appendage temperature. We placed a minimum bound on appendage temperature to keep leg tissues from freezing.

To simulate increasing leg temperatures caused by the shunting of venous blood flow to superficial veins, leg temperatures increase farther above ambient temperatures when the simulated animal is experiencing heat stress (cannot maintain its user-input activity levels without overheating). When this occurs, leg temperatures iteratively increase until the animal is no longer in heat stress, or until leg core temperatures reach a maximum value of the user-input core temperature for other (non-counter-current) body parts. This increase in appendage temperature occurs before any other (non-counter-current) body parts increase in temperature via the model’s other thermoregulatory mechanisms. Thus, the animal may cool off by losing heat to the environment through its legs before incurring any increases in core temperature of non-counter-current body parts.

To allow Whooping Cranes to wade, we modified Niche Mapper to incorporate input water type (salt or fresh water), the hours of the day during which an animal wades, the percentage of each hour that is spent wading (up to 100%), and hourly water depth, water temperature, and water velocity. The leg is then modeled as two separate body parts, the part of the leg below the water surface and the part of leg above the water surface, experiencing different environmental conditions. The lengths of the leg under water and above water are calculated based on water depth and leg length. The heat balance equation is solved separately for each of these portions of the leg. The portion of the leg in water loses heat to (or gains heat from) the water via convection, while the portion of the leg in air continues to exchange via convection and infrared and solar radiation. For an animal that wades while using counter-current heat exchange in its legs, the portion of the leg in water is set to a value above water temperature instead of air temperature.

### Time-energy Budgets During Doubly-Labeled Water Measurement Period

One model input is energy spent on activity during the animal’s active period for each day being modeled. Units are multiples of basal metabolic rate. Time-energy budgets by themselves can also serve as a method of estimating energy expenditure, particularly if the animal is in an environment where it does not experience energy costs of thermoregulation.

To obtain an estimate of energy spent on activity, we obtained a time-energy budget for each crane. Each crane was video-recorded as consistently as possible during all daylight hours of the 95.2 hour measurement period of the male and 95.0 hour measurement period of the female. (Videos were taken between the two blood draws because the doubly-labeled water technique produces a measurement of total energy expenditure specifically between these two draws.) Time budgets were extracted from the video tapes using an instantaneous sampling method at 30-second intervals. The same observer extracted data from all videos. Behaviors were assigned to the categories described in Table 1.The proportion of time spent in each behavior during each day was calculated after removing “out of sight” observations, where the bird was not captured on camera at the 30-second interval. Proportions of time spent in each behavior were calculated according to the equation

$$P_{i}=N_{i,day}/N_{tot,day}$$ (1)

where P_i_ is the proportion of the day spent in behavior i, N_i,day_ is the number of observations of behavior i for the day, and N_tot,day_ is the total number of in-sight behavior observations for the day.

**Table 1:** Energy costs assigned to behaviors for time-energy budget of two captive Whooping Cranes.

Behavior	Cost (xBMR)	Source
Rest–sleep^a^	1	Estimate
Rest–alert^b^	1.9	Lowest value of resting (perching) during the active period in [35]
Forage^c^	1.95	Middle of range of values in [35], as used by [36].
Walk	3.5	Estimate based on middle value for Marabou Storks on a non-sloped treadmill [37].^d^ ^,^ ^e^
Comfort^f^	1.95	Middle value for preening in [35], as used by [36]
Alarm call	1.15	Estimate based on cost of rooster crow in [38]
Unison call	1.15	Estimate based on cost of rooster crow in [38]
Dance	3	Estimate based on average cost of wing-flapping in [39]

Energy costs are reported as a multiple of basal metabolic rate (BMR).

^a^Sleeping with head drooping or resting on back.

^b^Resting but not sleeping.

^c^Food capture, manipulation, and consumption behaviors.

^d^Values in [37] reported as multiples of resting metabolic rate. Estimated xBMR value of rest-alert for Whooping Cranes used to convert this value to a multiple of BMR.

^e^ Similar to value estimated in [23] for pacing Whooping Cranes.

^f^Preening, stretching, bill flicking, head shaking, and head rubbing.

Behaviors were assigned to energy costs reported in the literature for other bird species [35,36,37,38,39] (Table 1), because the energy costs of different behaviors for Whooping Cranes have not been measured. Average daily energy expenditure on activity for each day (E_day_) was calculated as the average energy cost of activities, weighted by time spent in each activity:

$$E_{day}=\sum \left(P_{i}\times E_{i}\right)$$ (2)

where E_i_ represents energy cost of each behavior i (Table 1), and values are summed across all behaviors.

To check for any significant night-time activity, a trail camera was mounted on a tree, facing the pond in the Whooping Crane enclosure (where cranes were known to sleep), and set to take pictures every 5 minutes from dusk to dawn. Leg bands could not be seen in the resulting photographs and behaviors were difficult to discern from snapshots, but observers could watch for changes in location from one photograph to the next.

### Parameterization of Microclimate Model for Simulations of Doubly-Labeled Water Measurement Period

During the doubly-labeled water measurement period (the four days between the second and third blood samples), we collected data necessary to simulate Whooping Crane energy expenditure using Niche Mapper. Weather variables needed to simulate the microclimate conditions of cranes during each day of the study were measured using in a field adjacent to the Whooping Crane’s enclosure. Data were collected on a datalogger (Campbell Scientific 21x Micrologger) and transferred to a computer at the end of the experiment. Air temperatures and wind speed were collected using shielded thermocouples and 3-cup anemometers (Rimco) situated at 0.25m, 0.50m 1.00m, and 2.00m above the ground. Solar radiation in the horizontal plane was recorded using a pyranometer (Campbell Scientific CS300, range 300–1100 nm, measurement range 0–2000 W/m^2^). Water temperatures of the pond in the Whooping Crane enclosure were measured at three depths (approximately 1cm below the surface, 30cm below the surface, and 60cm below the surface) using thermocouples placed in the water and taped to a concrete barricade. All variables except wind speed were measured every 60 seconds, with 15-minute averages recorded by the datalogger. Temperatures were recorded to four significant digits. Wind speed was measured continuously, with total anemometer rotations recorded at 15-minute intervals. All thermocouples and anemometers were calibrated after the experiment. To obtain daily minimum and maximum humidity values, humidity was measured in an area adjacent to the Whooping Crane enclosure near sunrise and during early afternoon (between 13:00 and 15:00) with a Bacharach sling psychrometer.

Air temperatures and wind speeds were averaged by hour. Each day’s minimum and maximum hourly 2m air temperature and 2m wind speed were entered into the microclimate model. Measured humidity values were assumed to be similar to hourly averages. They were entered as each day’s minimum and maximum hourly values. Solar radiation was averaged by hour, and these hourly values were entered directly into the model. Water temperatures at 30 cm depth were also averaged by hour and entered directly into the model. No cloud cover was simulated because the sky remained clear during most of the study.

### Parameterization of Endotherm Model for Simulations of Doubly-Labeled Water Measurement Period

Morphometric and physiological input parameter values were collected to model each of the two captive Whooping Cranes studied using the doubly-labeled water technique. Body mass was obtained while Whooping Cranes were in hand for doubly-labeled water measurement procedures. In addition, torso diameter and length were measured using a tape measure. Depth (thickness) of the feather layer was measured using a ruler. Solar reflectivities of molted Whooping Crane feathers (from two different individuals) had previously been measured using an ASD portable spectroreflectometer (spectral range = 350–2500 nm).

Other morphometric measurements (lengths and widths of body parts) were obtained from still images captured from videos taken during the doubly-labeled water measurement period. We used the program ImageJ to measure lengths and widths of body parts in still images (http://imagej.nih.gov/ij/). Upper leg lengths (top of unfeathered leg to tibiotarsal joint) were scaled using the known height of the cranes’ metal leg bands. Other measurements were scaled using upper leg lengths. Within the model, adjustments to morphometric measurements of each body part were made when model-calculated densities of body parts (flesh only, without feathers) differed by more than 5% from 633 kg/m^3^, an estimate of plucked bird density based on unpublished lab measurements (Porter WP, unpublished). These adjustments were allowed because input body masses of cranes were measured directly, while most morphometric parameters were measured in photographs and thus subject to inaccuracies due to foreshortening and varying distances between different body parts and the camera.

While sleeping at night, Whooping Cranes usually stand on one leg with the other leg lifted and tucked into the ventral torso feathers. They also typically rest their heads and necks on their backs and tuck their beaks into their dorsal torso feathers. This sleeping posture changes the cranes’ surface area and corresponding heat exchanges with their environments. During hours 20:00–5:59, cranes were modeled standing on one leg, with head, neck, beak, and second leg tucked into the torso. Each crane’s shape was approximated as an ellipsoid incorporating the flesh volume of the torso, neck, head, beak, and second leg, and surrounded by a feather layer with the properties measured on the cranes’ torsos. This enlarged ellipsoid stood on a single cylindrical leg of unchanged size.

During collection of behavior data from videos for time-energy budget calculations, the depth of water (if any) in which each crane was wading was recorded along with behavior every 30 seconds. Water depths were recorded in relation to the length of the crane’s leg to the nearest of the following reference points: no water, water covering foot (toes) only, water half-way between foot and tibiotarsal joint, water up to tibiotarsal joint, water half-way between tibiotarsal joint and top of legs (bottom of torso), or water covering legs (reaching bottom of torso). Relative depths were converted to centimeters using known crane leg lengths. Average percent of time in water and average water depth while wading were calculated for each hour of the day and input into the model. At night, cranes were modeled standing in water of a depth half-way between the foot and tibiotarsal joint, based on observations from overnight trail camera photographs and direct observations of crane locations at the beginning and end of each day of the study.

Physiological input variables were obtained from the literature [40,41,42,43,44,45,46,47,48,49,50,51] and are summarized in Tables 2 and 3 along with the morphometric inputs.

**Table 2:** Animal model input parameters used to simulate energy expenditures of two captive Whooping Cranes (one adult male, one adult female) in outdoor and in metabolic-chamber-like conditions.

Parameter	Value	Source
Mass (kg)	5.05 (female) 6.15 (male)	Measured^a^
Fat mass (% body mass)	10	Estimate based on values in [40,41]^b^
Body parts with subcutaneous fat	Torso	Assumption that the majority of subcutaneous fat is stored in torso
Animal density (kg/m3)	633.3	Unpublished lab data from newly dead birds
Basal metabolic rate (W)	11.9 (female) 13.7 (male)	Allometric equation for non-passerine birds: Equation 3 in [42]^c^
Proportion of energy powering physical activity that is released as heat	0.8	[43] (General estimate for animals)
Core temp (°C)	40.7	Measured in [44] for Whooping Cranes
Max. core temperature (°C)	44	Estimate based on values in [45]^d^
Min. core temperature (°C)	37.7	A decrease of 1–3°C in body temperature is characteristic of most birds [46]
Solar reflectivity of feathers	0.62	Measured^e^
Solar reflectivity of legs and beak	0.33	Estimate^f^
Percent of skin acting as free water surface	0.2	Based on values used in [47]^g^
Thermal conductivity of flesh	0.5	Based on values measured in [48]
Maximum O_2_ extraction efficiency (%)	31	[49] (general value for birds)
Minimum O_2_ extraction efficiency (%)^h^	2.12 (female) 2.10 (male)	Allometric equation for birds panting: Equation 63 in [50]^i^
Configuration factor for infrared radiation: proportion of animal facing the sky	0.5	Estimate
Configuration factor for infrared radiation: proportion of animal facing the ground	0.3	Estimate

^a^Average of values measured on first and last days of doubly-labeled water measurements.

^b^Fat masses of Whooping Cranes are not available from the literature. The fat mass of the study animals was estimated as follows. A rough minimum boundary on possible fat content was also obtained based on annual October weights of the two study animals, which were available for the female and male for the 6 years and 7 years prior to the study, respectively. If the difference between their weight during the study and their lowest October weights were all fat, they would have at least and 8% (female) and 7% (male) body fat. Other northern-nesting crane species captive at the International Crane Foundation undergo fall weight gains between September and November [41]. However, our measurements were taken in September, when cranes would not have been gaining weight for long if the timing in their weight fluctuation is similar to that of other northern-nesting species. Thus, we assumed the study animals would have a minimal to moderate amount of fat. [40] found that Arctic-nesting Sandhill Cranes averaged 5% (male) and 7% (female) body fat early in spring migration and 23%-24% fat at end of their spring stopover period. 10% represents a moderate body fat percentage between these extremes.

^c^Body masses shown in this table were used.

^d^[45] found that body temperature of birds at an air temperture of 45°C was, on average, 3.3°C above body temperature at the lower critical temperature in a review of 28 studies.

^e^Reflectivities of molted Whooping Crane feathers measured using an ASD portable spectroreflectometer (spectral range = 350–2500 nm).

^f^See sensitivity analyses in main text.

^g^ Values for the Hawaiian Amakihi (*Hemignathus virens*) and the Hawaiian Anianiau (*Hemignathus parvus*) in [47] In this study, modeled water loss values were within ±2SE (standard error) of water loss values measured in metabolic chambers, except for one temperature point.

^h^Minimum O_2_ extraction efficiency is used to simulate panting (increase respiratory frequency without increasing the amount of oxygen absorbed into the body) when the modeled animal is under heat stress.

^i^Allometric Equation (63) in [50] gives f*pant*/f*rest* where f is respiratory frequency (per second) for panting and normal respiration and M is mass in kg. Normal oxygen extraction efficiency (see value in table) was divided by f*pant*/f*rest* for each individual.

**Table 3:** Morphometric input parameter values used to simulate energy expenditures of two captive Whooping Cranes (one adult male, one adult female) in outdoor and in metabolic-chamber-like conditions.

Parameter	Female	Male	Source
Feather element diameter (μm)	18.75	18.75	Measured on plastic-embedded ostrich skin with feathers
Feather element density (cm^-2^)	14400	14400	Measured on plastic-embedded ostrich skin with feathers^a^
Length of feathers on neck and head, d/v (mm)	25/24	22/26	Average of head and neck values measured on study animals^b^
Head feather depth, d/v (mm)	10/2	18/4	Measured on study animals^b^
Neck feather depth, d/v (mm)	10/10	10/9	Measured on study animals^b^
Torso feather length, d/v (mm)	60/60	61/55	Measured on study animals^b^
Torso feather depth, d/v (mm)	10/15	9/15^c^	Measured on study animals^b^
Head shape	Truncated cone		Approximation
Head length (cm)	10.3	10.6	Measured from photographs of study animals^d^.
Head diameter, proximal (cm)	7.0	6.8	Measured from photographs of study animals^d^. Average of vertical and horizontal diameters.
Head diameter, distal (cm)	3.4	3.5	Measured from photographs of study animals^d^. Average of vertical diameter and horizontal diameter.
Beak shape	Truncated cone		Approximation
Beak length (cm)	11.4	13.4	Measured from photographs of study animals^d^. From side view of head.
Beak diameter, proximal	3.4	3.5	Measured from photographs of study animals^d^. Average of vertical and horizontal diameter.
Beak diameter, distal	0.5	0.5	Estimate
Neck shape	Cylinder		Approximation
Neck length (cm)	31.5	29.7	Measured from photographs of study animals^d^.
Neck diameter (cm)	8.3	8.3	Measured from photographs of study animals^d^. Average value of middle and two ends of neck.
Torso shape	Ellipsoid		Approximation
Torso length (cm)	54.5	58.6	Estimated, based on measurements^e^
Torso diameter, vertical (cm)	24.0	21.6	Estimated, based on measurements^e^
Torso diameter, horizontal (cm)	20.0	19.6	Estimated, based on measurements^e^
Leg shape	Ellipsoidal Cylinder		Approximation
Leg length	37.5	35.9	Measured from photographs of study animals^d^. Length of unfeathered leg.
Leg diameter, front-back (cm)	1.7	1.7	Measured from photographs of study animals^d^. Average of diameter at middle of upper leg (top of unfeathered leg to tibiotarsal joint) and lower leg (tibiotarsal joint to foot).
Leg diameter, side-side (cm)	1.3	1.3	Measured from photographs of study animals^d^. Average of diameter at middle of upper leg (top of unfeathered leg to tibiotarsal joint) and lower leg (tibiotarsal joint to foot).

Abbreviations: d = dorsal, v = ventral.

^a^Density of fur or feathers has little effect on animal heat loss over a wide range of values [51].

^b^Measurements on study animals were made while the animals were in hand for doubly-labeled water injections (prior to injections) using tape measures and rulers.

^c^Due to incorrect reading of ruler, the female’s feather depth was used for both animals.

^d^Photographs were obtained by taking stills from time budget videos during the doubly-labeled water measurement period. The known height of the cranes’ leg bands were used to scale measurements of the lengths of the cranes’ unfeathered upper leg (tibiotarsal joint to bottom of feather line). Because bands were not visible in all photographs, the lengths of unfeathered upper legs were used to scale all other measurements. Measurements shown are an average of measurements from at least three photographs of each bird. To minimize effects of foreshortening, stills were in which the crane’s upper leg was in a vertical position and the crane’s front/back or side was perpendicular to the camera.

^e^The torso was modeled as an ellipsoid incorporating the volume of the wings and the feathered (top) portion of the legs. The wings were included because cranes’ wings are almost always folded against the body (except when flying, which is rare for captive cranes). The feathered part of the upper legs was included because feathers have a significant impact on heat balance, making this part of the leg more similar to the torso than the unfeathered portion of the legs. The following steps were taken to calculate the estimate the appropriate ellipsoid size. (1) For each crane, the length and circumference of the torso, including the wings, was measured with a tape measure. The ratio of horizontal to vertical diameter for the torso plus wings was obtained from photographs (0.98 for the male and 1.07 for the female). The ratio and circumference were used to calculate the horizontal and vertical diameter of the torso, according to the formula $C\approx \pi \left[3(a+b)-\sqrt{\left(3a+b)(a+3b\right)}\right]$, where C is the circumference of an ellipse, a is the semi-major radius, and b is the semi-minor radius. To obtain radii of a “flesh only” (i.e. unfeathered) ellipsoid, the dorsal and ventral feather depths were subtracted from the vertical diameter, and 4x the average of the dorsal and ventral feather depth were subtracted from the horizontal diameter. 4x feather depth was subtracted to account for the feathers on the outside of the ellipsoid and feathers between the wings and torso, which we assumed would be compressed by about 50%. 2x the average of dorsal and ventral feather depth was removed from the measured torso length. The volume of the “flesh only” ellipsoid was calculated using the formula $V=\frac{4}{3}\pi abc$ where V is volume, and a, b, and c are the radii. (2) The “flesh only” volume of the feathered portion of the legs was estimated using the formula for the volume of a truncated cone with constant semi-major to semi minor axis ratio: $V=\pi \frac{A}{B}\left\{\frac{B^{2}H_{t}^{3}}{3{\left[-BH_{t}/(b_{t}-B)\right]}^{2}}-\frac{B^{2}H_{t}^{2}}{\left[-BH_{t}/(b_{t}-B)\right]}{+B}^{2}H_{t}\right\}$ where A and B are the semi-major and semi-minor axes of the large (proximal) end of the truncated cone and H_t_ is the height of the truncated cone. The length of the feathered portion of the leg, diameter at the proximal end, and diameter at the distal ends were measured from pictures. The average of side-to-side and front-to-back diameters were used. Feather depth was assumed to be the average of torso dorsal and ventral depths and was subtracted from diameters to obtain A and B. (3) The volume of the “flesh only” feathered leg was added to the volume of the “flesh only” torso-plus-wings ellipsoid to obtain the “flesh only” volume for final ellipsoid. The “flesh only” diameters of the new ellipsoid were calculated from the formula for the volume of an ellipsoid (above), maintaining the same a:b and b:c ratios as the torso-plus-wings “flesh only” ellipsoid. Torso feather depths were then added to diameters of the torso-plus-wings-plus-feathered-leg “flesh only” ellipsoid. 2x the average of the dorsal and ventral feather depths were added to the horizontal diameter, and the sum of the dorsal and ventral feather depths was added to the vertical diameter.

Parameters needed to model crane legs with counter-current heat exchange (minimum difference between ambient temperature and average leg temperature, minimum bound on average leg temperature) were estimated using data from thermal images. Photographs of the cranes were taken opportunistically throughout the doubly-labeled water measurement period using a thermal imaging camera (FLIR T360). A “blackbody” of known temperature was included in the images to calibrate the camera. The blackbody was a metallic object painted with black spray paint, which has high emissivity in the infrared spectrum. A thermocouple was attached to the blackbody and connected to a data logger (Campbell Scientific 21x Micrologger). Leg temperatures were measured using the program FLIR ResearchIR. Input parameters to the software include thermal emissivity, which we assumed to be high (0.98) for the crane’s legs, as is usual for biological surfaces (e.g. values in Campbell and Norman [52], Table 11.3.) An emissivity of 0.98 was also used for the blackbody reference plate. Humidity and air temperature at the time of the photograph were calculated from measured microclimate variables, and distances of cranes and the blackbody from the camera were estimated. Atmospheric long wavelength infrared transmission was assumed to be 0.99. Average leg temperatures were obtained by taking the average pixel value along a line drawn lengthwise down the crane leg. When measuring a leg with a metal leg band, the average leg temperature was measured above and below the band. A weighted average of the two values was calculated based on the relative lengths of the two lines. The temperature of the blackbody was measured by averaging the values of pixels within a box drawn on its image. The difference between the temperature measured by the camera and the temperature recorded by the thermocouple was used to correct Whooping Crane leg temperature in each photograph. (One to four legs were measured per image, depending on whether both individuals were in the image and whether both legs were visible on each individual).

The energy expenditure of each crane was simulated across all four days of the study. Cranes were modeled as active during hours with solar radiation (15-hour periods) and inactive during periods without solar radiation (9-hour periods). The length of the activity and rest periods corresponded well with direct observations and overnight trail camera photographs of the cranes (see Whooping Crane Time-Energy Budgets during Doubly-Labeled Water Measurements subsection of Results).

During active hours, the energy expenditure on activity for each crane was set equal to the energy expenditure calculated from its daytime time-energy budget for that day (daily energy expenditures during active hours in Table 4). This activity-energy value can also be thought of as a “target metabolic rate” for the model. That is, thermoregulatory mechanisms engaged if heat production greater than that produced by time-energy-budget-based activity levels was required to maintain the crane’s core body temperature or if the crane was unable to maintain its activity level without overheating.

**Table 4:** Average daily energy expenditure on activity for two captive Whooping Cranes (one male, one female) during a four-day period over which energy expenditure was measured using the doubly-labeled water technique.

	Energy expenditure (xBMR) during active hours		Energy expenditure (xBMR) including rest (night) hours	
Date	Female	Male	Female	Male
9/24/2014	2.34	2.25	1.8	1.8
9/25/2014	2.47	2.51	1.9	1.9
9/26/2014	2.46	2.48	1.9	1.9
9/27/2014	2.33	2.36	1.8	1.8
9/28/2014	2.33	2.39	1.8	1.9
Avg.	2.39	2.39	1.9	1.9
St.dev	0.07	0.12	0.0	0.1

Energy expenditures are expressed as multiples of basal metabolic rate (BMR). Energy expenditures during active hours were calculated from time budgets observed during daylight hours (sunrise to sunset) on each day of the study. The five daily values for each individual were used as model input, with cranes modeled as diurnal and crepuscular (i.e. active while there is solar radiation in their location), and assuming a value of 1xBMR during hours without solar radiation (nine hours per night). Energy expenditures including rest (night) hours are shown for reference only (not model input) and were calculated assuming a metabolic rate of 1x BMR during a nine-hour rest period. The nine-hour rest period is consistent with observed rest times based on photographs taken overnight by a trail camera in the crane enclosure.

During the rest (night) period, cranes were modeled with a “target” metabolic rate equal to basal metabolic rate. That is, thermoregulatory mechanisms engaged if a metabolic rate higher than basal metabolic rate was required to maintain the crane’s core body temperature, or if the crane was unable to maintain its basal metabolic rate without overheating.

The activity multipliers used were calculated from time-energy budgets, rather than doubly-labeled water measurements, for two reasons. First, the doubly-labeled water method produces average energy expenditure values for the total duration of the measurement period. However, animals engage in different activities with different associated heat production during active and rest periods, during which they also experience different microclimates. We used the finer-scale data from the time-energy-budget analysis in order to more accurately simulate heat exchanges between the cranes and their environments. Second, we wished to compare the model and doubly-labeled water technique as two independent measurements of energy expenditure.

For comparison of simulated energy expenditure values to doubly-labeled water results, the average simulated energy expenditure across all hours of the study (between the two blood draws following doubly-labeled water injection) was calculated for each crane. The average energy expenditure across the two individuals was also calculated.

### Testing Modeled Leg Temperatures

To test the model’s ability to accurately simulate Whooping Crane leg temperatures, we modeled average Whooping Crane leg skin temperatures under the microclimate conditions measured at the crane enclosure at the time that each photograph was taken.

Cranes were simulated as described in the Parameterization of Endotherm Model for Simulations of Doubly-Labeled Water Measurement Period subsection with the following exceptions. Hourly air temperature, water temperature, and solar radiation values were replaced with measured values interpolated from 15-minute readings to the minute that the photograph was taken. Average 15-minute wind speeds, including the minute that the photograph was taken, were used to replace hourly average wind speeds. Wind speeds at 1m height (rather than 2m height) were used to simulate wind conditions closer to leg height. For photographs in which cranes were wading, water depth specific to each photograph was used. Water depths were estimated to the nearest reference point described in the Parameterization of Endotherm Model for Simulations of Doubly-Labeled Water Measurement Period subsection. For photographs in which cranes were not standing in water, infrared photography-measured leg temperatures are compared to the simulated average leg core temperature for the whole leg. For photographs in which cranes were wading in water, infrared-photography-measured leg temperatures are compared to simulated average leg core temperatures for the portion of the leg above the water surface. Infrared photography-measured temperatures measured on legs held above the water during periods when the bird was resting on the other leg were not used because the model does not currently simulate this posture for cranes. All visible legs in infrared photographs were used (one to four legs per photograph).

### Metabolic Chamber Simulations

For the metabolic chamber simulations, microclimate conditions included low wind speed (0.01 m/s), low relative humidity (5%), no solar radiation, and ambient temperatures ranging from -50°C to 50°C in 1°C intervals. To explore the effects of wading on heat loss, cranes were simulated in water of varying depths, with water temperature matching air temperature. Simulated water temperatures were held constant at 0.1°C when air temperatures were less than or equal to 0°C. Crane physiological and morphological parameters were the same as those used for simulations of energy expenditure for comparison to doubly-labeled water measurements.

### Sensitivity Analyses

Sensitivity analyses were carried out in order to test the sensitivity of the Whooping Crane energy expenditure model to several morphological, physiological, and environmental input parameters. Whooping Cranes were modeled in metabolic-chamber-like conditions of low wind speed (0.01 m/s), low relative humidity (5%), and ambient air temperatures that changed in 1°C intervals. Solar radiation was set to 0 W/m^2^ except when testing effects of changing animal solar reflectivity and of solar radiation itself. For testing the effects of changing animal solar reflectivity, solar radiation was set to 334.1W/m^2^, half the maximum value measured during the doubly-labeled water study.

Air temperatures from -25 to 35°C were modeled to approximate the range of air temperatures that migratory Whooping Cranes experience in the wild as well as the smaller range experienced during doubly-labeled water measurements in this study. This temperature range was estimated based on the air temperatures occurring at breeding grounds and wintering grounds of the two existing migratory populations of Whooping Cranes [53], as described in S3 Text.

For these analyses, all thermoregulation mechanisms except for counter-current heat exchange in legs were turned off, so the parameter being tested would be the only value changing with air temperature. Counter-current heat exchange in legs was allowed due to its effect on metabolic rate and in order to examine effects of other variables in the context of realistic leg temperatures and metabolic rates for cranes. Specifically, we tested the effects of the following biological variables: feather element diameter, feather element density, feather length, feather layer depth, body fat content, morphometry (length and width of body parts), solar reflectivity of entire body, solar reflectivity of legs, core temperature, minimum difference between leg core temperature and air temperature, and minimum difference between leg core temperature and water temperature for a crane with legs entirely submerged in water. We also tested the effects of the environmental variables of wind speed and solar radiation.

Input variables were changed by ±10% so that relative effects could be compared between variables of different units. Additional variation in individual input variables were also modeled based on possible real variation in Whooping Crane biology or environmental conditions. In particular, feather properties were varied by ±50% to examine effects over a wide range of values. Feather layer depth was varied up to +100%. Body fat values were varied from 1%, an extreme minimum value, to 20%, a maximum based on values measured for Sandhill Cranes at staging areas during spring migration in [40]. Because leg solar reflectivities were not measured directly on Whooping Cranes, we varied these values up to minimum and maximum values available for reptile skins in our lab database (34 species in a variety of colors, including black and white; Porter WP, unpublished). Body core temperatures were varied to the minimum and maximum values allowed in the model for thermoregulation. The minimum difference between leg temperature and ambient temperature was increased up to 10°C. Wind speeds were varied from 0m/s to the average daily maximum value measured for doubly-labeled water measurement period. Solar radiation values were varied from 0 W/m^2^ to the average daily maximum measured for the doubly-labeled water measurement period, 668.2 W/m^2^.

## Results

### Microclimate During Doubly-Labeled Water Measurements

Measured microclimate variables during the doubly-labeled water period are summarized in S1–S4 Figs Water temperatures at 30cm depth were used to estimate average water temperature along the length of the leg because temperatures at 30cm depth were very close to water surface temperatures (average difference of 0.18°C, range of differences -0.34°C to 2.4°C), and Whooping Crane leg lengths were not much longer than 30cm (37.5 cm and 39.5cm). The difference between 39.5cm water depth and 30cm water depth (assuming linear temperature change between 30cm and 60cm water depth temperature) was small (range -1.12°C to -0.24°C) and would have little effect on average water temperature along the length of the leg.

### Whooping Crane Time-Energy Budgets During Doubly-Labeled Water Measurements

Trail camera photographs indicated that, although some night-time changes in location occurred over periods up to 30 minutes long, cranes were stationary in the pond for most of each night, starting at a time between about 20:30 and 22:00. Cranes began changing location regularly between 6:00 and 6:30 in the morning

The overall proportion of time that the cranes were out of sight during the daytime was low (5.8% for the female and 12.9% for the male). Time spent in different behaviors during daylight hours is shown in Table 5. Table 4 shows daily energy expenditures calculated from these time-activity budgets. Energy expenditures during active hours were larger than energy expenditures including rest hours because a nine-hour nocturnal period at basal metabolic rate (BMR) was incorporated into the latter. These smaller values estimate daily energy expenditure for the Whooping Cranes if no energy costs of thermoregulation were being incurred.

**Table 5:** Percent daily time spent in different behaviors by two captive Whooping Cranes (one adult male, one adult female) during daylight hours during a four-day doubly-labeled water measurement period.

Crane	Date	Comfort^a^	Foraging^b^	Walking	Rest-Alert^c^	Rest-Sleep^d^	Social^e^
Female	9/24	24.5	9.4	28.1	35.0	2.9	0.0
	9/25	16.9	12.0	35.5	34.5	1.0	0.0
	9/26	19.9	9.6	34.2	35.5	0.4	0.4
	9/27	19.2	9.4	25.8	45.5	0.0	0.1
	9/28	26.9	15.2	25.7	32.0	0.0	0.2
	Avg.	21.5	11.1	29.9	36.5	0.9	0.1
Male	9/24	28.4	15.6	18.4	36.7	0.9	0.0
	9/25	10.4	20.5	37.5	30.6	0.9	0.0
	9/26	7.1	28.6	35.4	28.7	0.0	0.2
	9/27	14.2	21.7	27.5	36.4	0.0	0.2
	9/28	26.8	7.8	29.5	35.4	0.0	0.4
	Avg.	17.4	18.8	29.7	33.6	0.4	0.2

^a^Preening, stretching, bill flicking, head shaking, and head rubbing.

^b^Food capture, manipulation, and consumption behaviors.

^c^Resting but not sleeping.

^d^Sleeping with head drooping or resting on back.

^e^Dancing, alarm calling, unison calling.

On average, the two cranes spent similar amounts of energy on activity, when normalized to basal metabolic rate (Table 4) due to similar time budgets and similar energy costs of comfort, foraging, and daytime resting behaviors. (See S2 Text for detailed analysis of behaviors in time budgets.) However, the average amount of energy spent on activity was lower for the female crane (1891.8 kJ/day on average compared to 2201.6 kJ/day for the male crane) due to the female’s lesser weight and, thus, lower estimated basal metabolic rate.

### Measured Leg Temperatures

**Fig 2:**
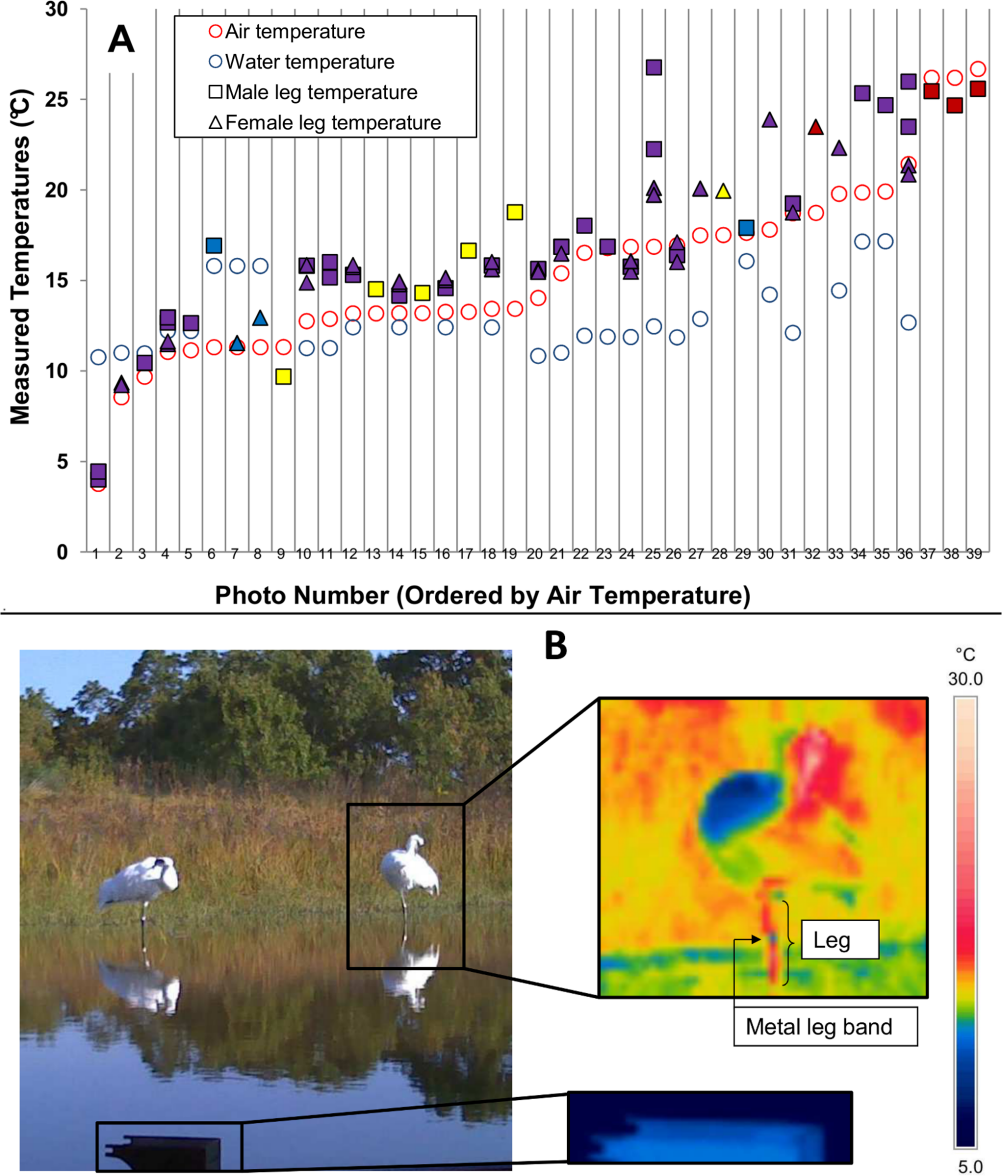
The average leg temperatures of two captive Whooping Cranes (one male, one female) measured from infrared photographs taken during a five-day period over which energy expenditure was measured. (A) Average leg temperatures of the two Whooping Cranes in each thermal image, along with local air temperatures and pond water temperatures (for images in which birds were wading) at the time the image was captured. X-axis values are categorical, with each column in the chart representing data from a given image. Images were taken across four days at different times of day, but are arranged in order of increasing air temperature (from left to right) for clarity. Shapes of data points represent the individual on which the leg temperature was measured (square = male, triangle = female). Colors of data points represent whether the bird was wading and the portion of the leg visible to the camera. Purple points represent values from leg visible above the water surface when birds were wading in water of depth approximately half-way between the foot and tibiotarsal joint. Blue points represent values of the leg visible above the water surface in shallow water, with depth less than one-quarter of the distance between the bird’s foot and tibiotarsal joint. Yellow represents temperatures measured on a leg held above the water during periods when the bird was resting on the other leg, with values measured from the tibiotarsal joint to the foot (the only portion of the leg that was usually visible when birds rested in this position.) Red points represent measurements from the entire length of the leg when birds were not wading. One to four legs were measured per image, depending on the number of legs visible to the camera from the two individuals. Air temperatures were interpolated linearly to the minute from averages recorded every 15 minutes from a shaded thermocouple placed outside the crane enclosure at 2m height. Pond water temperatures were measured using a thermocouple placed at 30cm depth at the deep edge of the cranes’ wading pond. Temperatures at 30cm depth were very similar to temperatures at shallow depths (S2 Fig). (B) Portions of an infrared photograph from which Whooping Crane leg temperature was measured, including a Whooping Crane and the temperature reference plate (lower left; boxed digital image and zoomed is the IR view) used to calibrate the thermal image, along with a digital image taken simultaneously by the camera. The temperature scale is shown on the right. Fig 2 and all photographs therein are original to this study.

Average temperatures of the visible portion of the Whooping Cranes’ legs were measured in 31 images, for a total of 70 values. Fig 2 shows the average leg temperatures from each image (squares for male’s legs and triangles for female’s legs, with colors indicating how much of the leg was visible above the water surface), along with air temperatures (red circles) and pond water temperatures (blue circles) at the time each image was taken. Overall, Whooping Crane leg temperatures were positively correlated with air temperature (R^2^ = 0.82), as expected for bird legs with counter-current heat exchange mechanisms. For legs that were partially submerged in water, air temperatures explained more variation in visible leg temperature (R^2^ = 0.82) than did water temperature (R^2^ = 0.20). Air temperature varied more than water temperature throughout the days of the experiment (S1 Fig and S2 Fig). When air temperatures fell below water temperatures, the average temperature of the visible portion of the leg tended to drop below water temperature (Fig 2). Leg temperature continued to increase with air temperature, even as air temperature increased above water temperature. Neither individual had consistently higher or lower leg temperatures than the other individual.

Because Whooping Cranes were able to keep measured leg skin temperatures within 1°C of air temperature even at the coldest measured air temperature (3.8°C), leg core temperatures were modeled at 1°C above environmental (air or water) temperature in Niche Mapper simulations.

We assumed that measured skin temperatures and modeled core temperatures would be comparable because skin and core temperatures should not differ much for crane legs due to their composition (primarily tendons and bone, which tend to have low metabolic rates compared to tissues found in other body parts, e.g. kidneys, heart; [54]) and small diameter (1.3 to 1.7 cm). When air temperatures were warmer than water temperatures, the distal (under water) portion of Whooping Crane legs was modeled as cooler than the proximal portion. When air temperatures were cooler than water temperatures, the measured average temperature of the visible portions of legs dropped below water temperature, but leg temperatures under water were unlikely to remain this cool when surrounded by warmer water. Thus, modeled leg temperatures under water were allowed to be higher than leg temperatures above water, under these conditions.

Simulated leg temperatures were allowed to increase further above environmental temperatures (up to a maximum of 40.7°C, the simulated core temperature of other body parts) when the modeled cranes experienced heat stress. A minimum boundary on leg simulated temperature was set at 3°C to keep the appendage above freezing temperatures. The same counter-current exchange temperature parameters were used to model crane beaks.

### Comparison of Metabolic Rates from Different Methods

Table 6 shows the average daily energy expenditures modeled using Niche Mapper, estimated based on time-energy budgets alone, and measured using the doubly-labeled water technique. (See S3 Table for the isotope concentrations measured in crane blood samples.) For each method of estimating or measuring energy expenditures, the female’s energy expenditures were smaller than the male’s. For each individual, all three values of average daily energy expenditure fell within a range of approximately 150 kJ/day (Table 6). Values averaged across the two individuals fell within a range of 23.4 kJ/day.

**Table 6:** Average energy expenditures (EE) of two captive Whooping Cranes (one adult male, one adult female) over a four-day period measured using the doubly-labeled water (DLW) technique, time energy budgets, and Niche Mapper (which incorporates the time-energy budgets from Table 4).

Individual	EE measured by DLW (kJ/day)	EE from time-energy budgets (kJ/day)	Niche Mapper-modeled EE (kJ/day)
Female	1810.9 (1.8x)	1953.5 (1.9x)	1929.7 (1.9x)
Male	2380.6 (2.0x)	2249.0 (1.9x)	2227.9 (1.9x)
Average	2095.8 (1.9x)	2101.2 (1.9x)	2078.8 (1.9x)

Values in parentheses are energy expenditures expressed as multiples of basal metabolic rate (See Table 2 for basal metabolic rates).

For the male, doubly-labeled water-measured energy expenditure was slightly larger than the time-energy budget estimate (+131.6 kJ/day). For the female, doubly-labeled water-measured energy expenditure was slightly smaller than the time-energy budget estimate (-142.6 kJ/day).

Niche Mapper did not simulate energy costs of thermoregulation above heat energy generated by activity or indicate that simulated cranes could not maintain their observed levels of activity without overheating. For both individuals, Niche Mapper-modeled energy expenditures fell within 5% of average daily energy expenditures predicted based on time-energy budgets, which were also input in to the model to simulate energy expenditure on activity. Niche Mapper-modeled output values were not exactly equal to activity-energy inputs. The slight differences occurred because the model ceases to engage in further thermoregulatory mechanisms when modeled hourly energy expenditures fall within 5% of minimal values needed for activity and basal functions. This small error range is needed to prevent the model from entering infinite loops. That is, Niche Mapper did not simulate additional energy costs of thermoregulation above time-activity budget levels or indicate that the cranes could not maintain their observed levels of activity without overheating.

**Fig 3:**
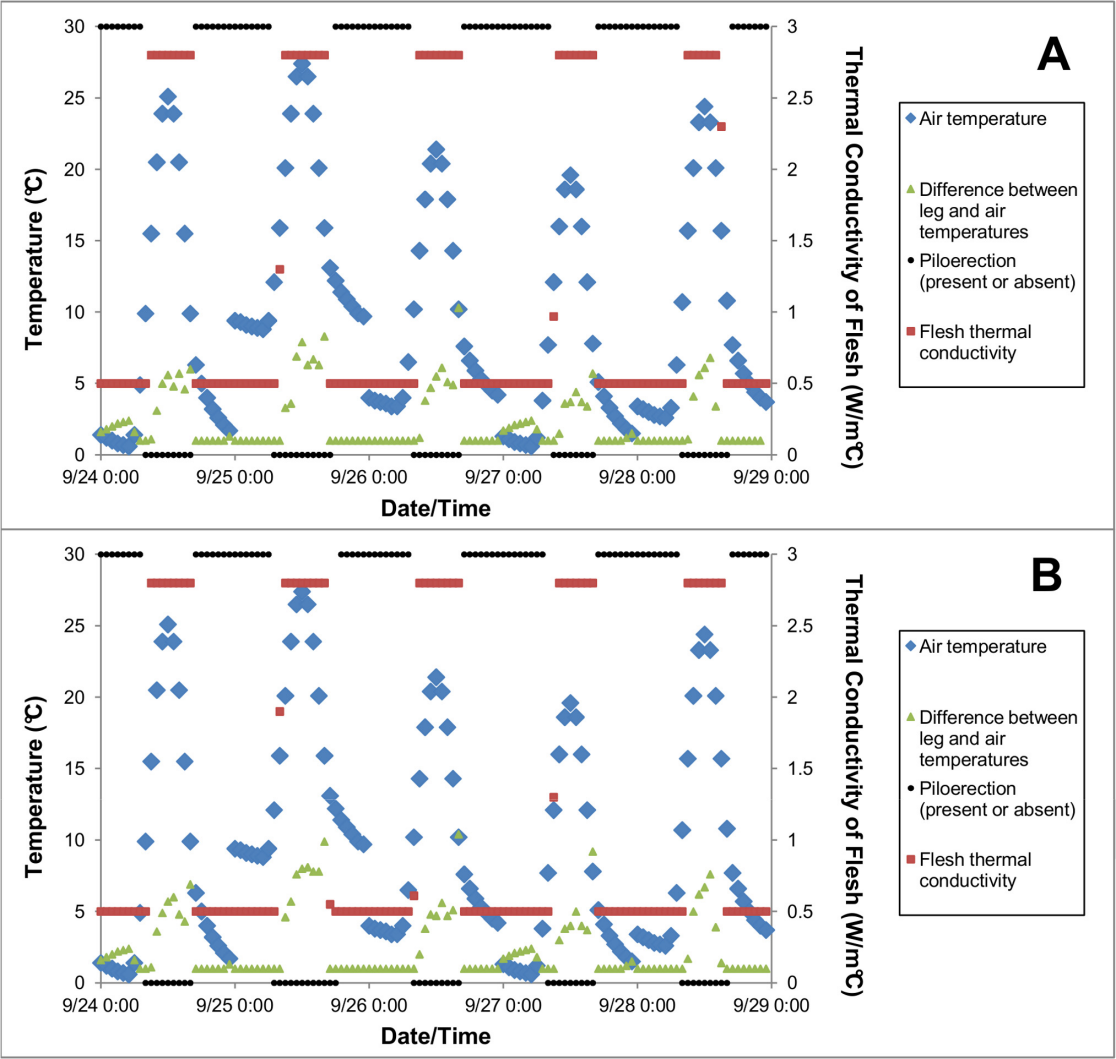
Thermoregulatory mechanisms used in model simulations of the energy expenditure of two captive Whooping Cranes (one male, one female) across five days during which their energy expenditures were also measured using the doubly-labeled water technique. Metabolic rates modeled using morphological and physiological properties of (A) the female crane and (B) the male crane measured in this study.

However, modeled cranes did engage in low energy-cost thermoregulatory mechanisms. That is, the model needed to simulate these thermoregulatory mechanisms to minimize crane metabolic rate during cooler periods of the study and to prevent overheating during warmer periods of the study. Ptiloerection was simulated during cooler periods of the study. Vasodilation and reduced use of counter-current blood vessels (increased difference between leg and ambient temperatures) were simulated during warmer periods of the study. Fig 3 shows the changes in flesh thermal conductivity (vasodilation), difference between air and leg temperature, and ptiloerection that occurred for each crane in model simulations. Simulated cranes engaged in ptiloerection in the evening, overnight, and early morning, when ambient temperatures were cooler and targeted heat production was lowest. Simulated flesh thermal conductivity increased (simulating vasodilation) to maximum values during the middle of the day, followed by simulated increases in the difference between leg temperature and ambient temperature (simulated reduced use of counter-current blood vessels). The model did not predict any panting or change in core temperature.

### Testing Modeled Leg Temperatures

**Fig 4:**
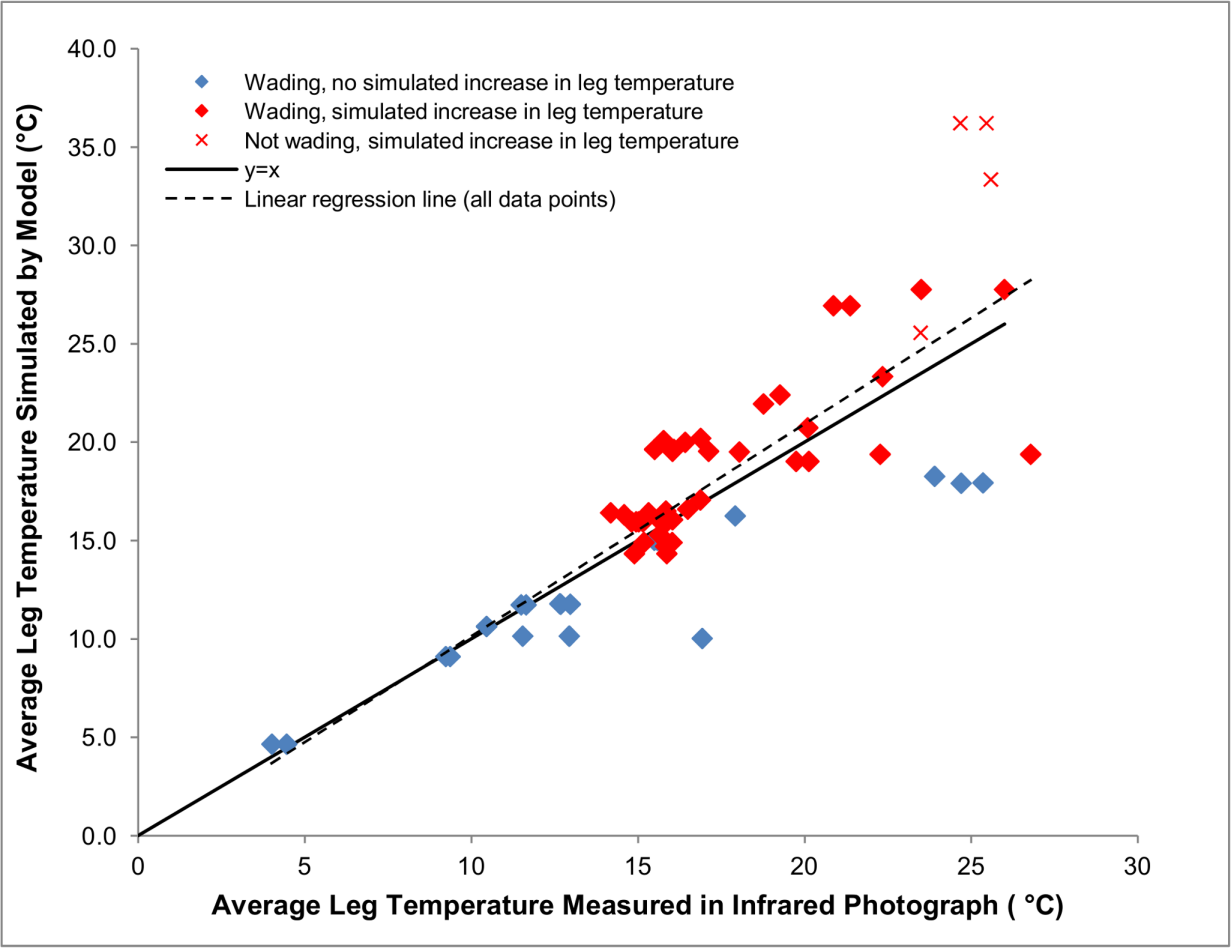
Average modeled and infrared-photography-measured leg temperatures of two captive Whooping Cranes (one male, one female). Leg core temperatures were modeled at 1°C above air temperature and allowed to increase farther above air temperature to prevent overheating. Cranes were modeled as wading in water or standing out of water to match conditions in the photographs. Red color indicates that the model simulated increases in leg temperatures to greater than 1°C above air temperature. The line showing y = x is shown for reference. The equation of the dotted line of best fit through the data points is y = 1.078x − 0.6401 (R^2^ = 0.699, n = 64).

Fig 4 shows leg temperatures simulated by the model under the microclimate conditions measured during the minute in which each photograph was taken. A linear regression of modeled leg temperatures against infrared-photograph-measured leg temperatures produced an R^2^ value of 0.699 (n = 64 legs). The model overestimated three leg temperatures from two photographs by particularly large extents, 8–12°C. These points occurred for non-wading cranes at the highest air temperatures during which photographs were taken. Overall, 66% of modeled data points fell within ±3°C of their corresponding infrared-photograph-measured temperatures, and 81% fell within ±6°C.

### Metabolic Chamber Simulations

**Fig 5:**
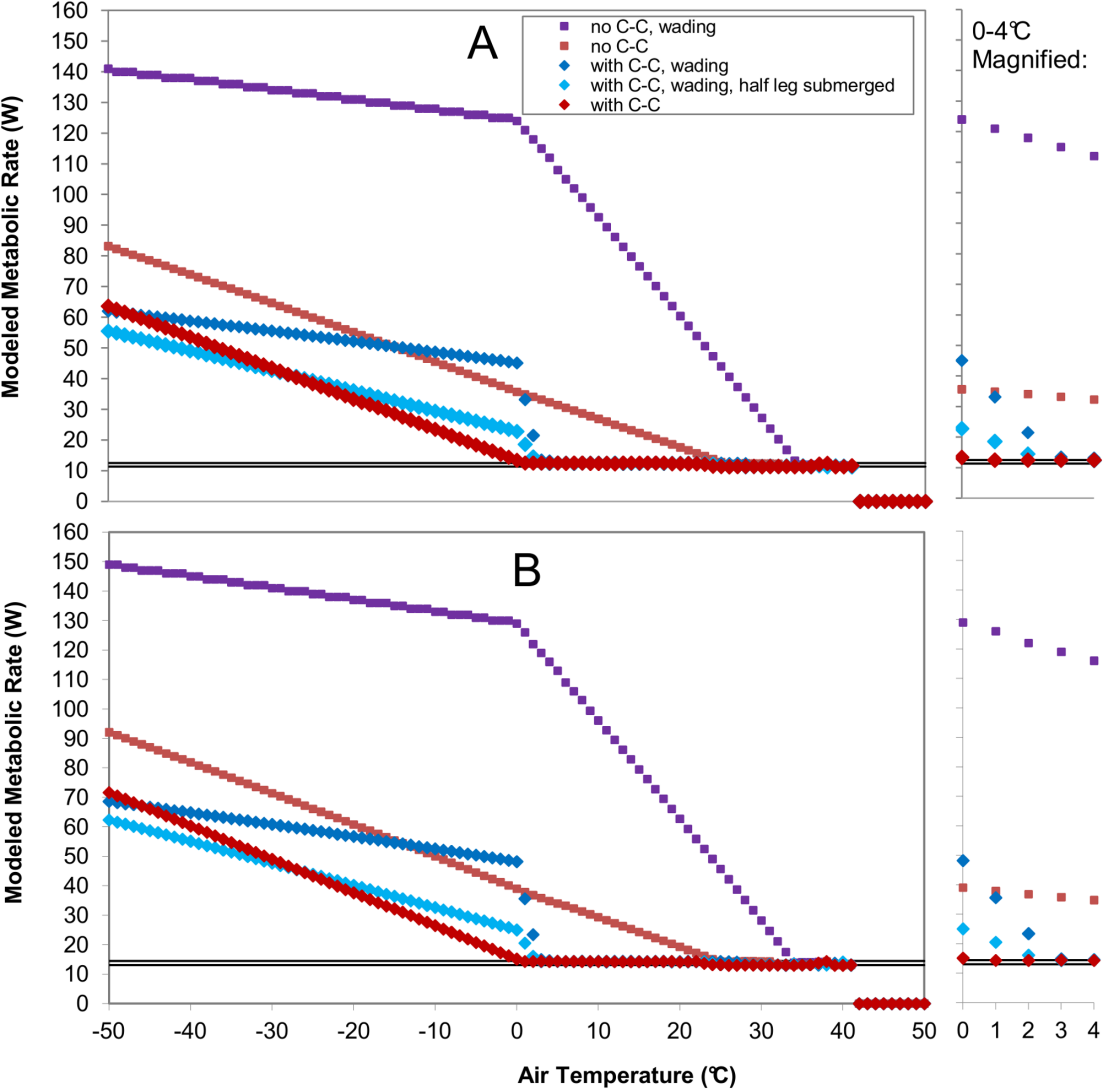
Modeled metabolic rates for two Whooping Cranes across a range of air temperatures in metabolic-chamber-like environmental conditions, with and without legs submerged in water (“wading”), and with and without counter-current heat exchange (“C-C”) in legs. Metabolic rates modeled using morphological and physiological properties of (A) the female crane and (B) the male crane measured in this study. Horizontal lines show ±5% of basal metabolic rate (Table **2**) for each crane. The model treats metabolic rates within this range as basal metabolic rate. (See Methods section of main text for explanation.) Legs submerged in water are completely submerged unless otherwise noted in the key. Water temperatures were set equal to air temperatures down to a temperature of 0°C (ice water), below which water would freeze. When counter-current heat exchange in legs was modeled, leg temperatures were set to 1°C above ambient temperature. Leg temperatures were allowed to increase farther above air temperature for thermoregulation when the model indicated heat stress. A minimum value of 3°C and maximum value of core temperature for other body parts (40.7°C) was set to prevent unrealistically high or low (freezing) leg temperatures.

Fig 5 shows the modeled metabolic rates of Whooping Cranes across the range of ambient temperatures in metabolic chamber-like conditions. Model results with and without counter-current heat exchange in legs and with legs submerged in water are also shown for comparison. While the male crane generally had a higher metabolic rate due to larger body size, metabolic rates showed the same patterns of variation with changing ambient temperature.

Metabolic rates for non-wading cranes with counter-current heat exchange in legs (red points in Fig 5) changed as expected with ambient temperature. At low temperatures, change in modeled energy expenditures with air temperature occurred as predicted by the Scholander-Irving model [55]. Modeled metabolic rate decreased approximately linearly with increasing ambient temperatures until the thermoneutral zone was reached. Because Niche Mapper treats metabolic rates within a small range (±5%) of basal metabolic rate as equivalent to basal metabolic rate when simulating low-energy-cost thermoregulatory mechanisms (e.g. ptiloerection, vasoconstriction), the predicted zone occurs where modeled metabolic rates fall within the horizontal lines delineating ±5% of basal metabolic rate in Fig 5. The lower critical temperature for both modeled Whooping Cranes was 1°C. Average values of minimal conductance (heat loss divided by the difference between deep body temperature and ambient temperature, when ambient temperatures lie below the thermoneutral zone; [56]) were 0.51 W/°C for the female crane and 0.57 W/°C for the male crane over modeled air temperatures of -25 to 0°C, the range of temperatures below the thermoneutral zone that Whooping Cranes are likely to experience in the wild.

At high ambient temperatures, modeled metabolic rates did not match the Scholander-Irving model [55] because energy costs of panting are not simulated in Niche Mapper. Instead, metabolic rates remain in the thermoneutral zone with increasing ambient temperature until the animal can no longer maintain its basal metabolic rate without overheating. Above this point, the declining metabolic rates that could be maintained without increasing core temperature are shown. The upper critical temperature for both modeled Whooping Cranes, where low-energy-cost thermoregulatory options were no longer sufficient and panting was required to maintain core temperature, occurred at 36°C.

When metabolic rates for Whooping Cranes with legs submerged in water were modeled (dark blue points in Fig 5) the “lower critical temperature” increased to 7°C for the female and 5°C for the male. Wading increased the “upper critical temperature” to 41°C for both individuals. (Note that true lower and upper critical temperatures do not incorporate wading.) These changes occurred because water has a higher convective heat transfer coefficient than air of the same temperature and velocity.

As ambient temperature began to decline below the lower critical temperature, metabolic rate increased very gradually, increasing only about 1W. Then, at 2°C, metabolic rates began to increase more and more rapidly, through 0°C. This steep increase occurred because Whooping Crane leg temperature had reached the minimum allowed temperature (3°C) and the difference between water and leg temperature was increasing, causing large increases in convective heat loss due to water’s large heat transfer coefficient. Because water temperature was not allowed to decline below 0.1°C, the difference between leg and air temperature remained constant at air temperatures below 0°C. This led to a slower rate of increasing metabolism with decreasing temperature in wading simulations compared to non-wading simulations. The smaller, constant temperature gradient between the legs and water below 0°C (compared to the steadily increasing one between the legs and declining air temperature) offset the effect of the greater convection heat transfer coefficient of water. However, savings in overall energy cost of thermoregulation by standing in ice water were not achieved until temperatures were very cold (less than -40°C).

Patterns of modeled metabolic rates for simulated Whooping Cranes with half the length of their legs submerged in water (light blue points in Fig 5) were intermediate between simulations with legs fully submerged and simulations without water, as expected. Lower critical temperatures increased to 3°C for both individuals, and upper critical temperature increased to 40°C for both individuals. A steep increase in metabolic rate occurred between 0°C and 2°C, but the rate of increase was not as steep as that of legs fully submerged in water. The rate of change in metabolic rate as air temperature decreased below 0°C was intermediate between the fully submerged leg and the leg in air. Savings in overall energy cost of thermoregulation by standing in ice water would still not be achieved until temperatures were colder than -40°C.

When counter-current exchange was not modeled in Whooping Cranes legs, the thermoneutral zone was much narrower, as expected (pink points in Fig 5). The lower critical temperature without counter-current exchange increased to 26°C and 25°C for the female and male, respectively. The “lower critical temperature” while wading increased even further (as expected) to 35°C and 34°C for the female and male (purple points in Fig 5).

### Sensitivity Analyses

Table 7 shows changes in model output in response to varying parameter values by ±10%. Additional (wider) variation in values is shown in S8–S10 Figs When biological variables were varied by ±10%, the model was most sensitive to changes in morphometric dimensions (length and width of body parts) (Table 7). In general, modeled metabolic rate increased with animal size. This increase is expected when environmental temperatures are below skin temperatures due to increased surface area from which heat can be lost to the environment via convection and net infrared radiation. The rate of increase in metabolic rate with declining temperature was larger for larger animals (as expected for metabolic rates that have not been normalized to body mass). This led to a slightly greater magnitude of change in metabolic rate when size was increased by 10% compared to when it was decreased by 10%.

**Table 7:** Average changes in simulated metabolic rates (W) of Whooping Cranes in metabolic-chamber-like environmental conditions across a range of air temperatures (-25°C to 35°C^a^) in response to changes in various environmental, physiological, and morphological model parameters.

Input parameter	Increase in parameter value	Change in output metabolic rate (W) (male/female)	Decrease in parameter value	Change in output metabolic rate (W) (male/female)
Morphometric dimensions	+10% of original value	6.2/6.3	-10% of original value	-5.7/-5.7
Solar reflectivity (all body parts)	+10% of original value	1.0/1.2	-10% of original value	-1.0/-1.2
Solar reflectivity of legs only	Increase to 52%^b^	1.6/2.0	Decrease to 4%^c^	-2.5/-3.1
Feather layer depth	+10% of original value	-0.4/-0.2	-10% of original value	0.5/0.8
Feather length	+10% of original value	0.2/0.2	-10% of original value	-0.1/-0.1
Feather element density	+10% of original value	0.3/0.4	-10% of original value	-0.3/-0.3
Feather element diameter	+10% of original value	0.8/0.9	-10% of original value	-0.7/-0.7
Fat content (% body weight)	+10% of original value	-0.03/-0.04	-10% of original value	0.03/0.04
Core temperature	Decrease to 37.7°C^d^	-1.8/-2.1	Increase to 41.7°C^e^	0.6/0.7
Minimum difference between leg temperature and air temperature	Increase to 3°C Increase to 10°C	1.4/1.6 4.2/6.8		
Minimum difference between leg temperature and water temperature^f^	Increase to 3°C Increase to 10°C	7.3/7.5 28.4/31.9		
Incoming solar radiation	+10% of original value	-2.8/3.2	-10% of original value	2.8/3.2
Wind speed	+10% of original value	0.3/0.5	-10% of original value	-0.3/-0.3

Metabolic rates are modeled using morphological and physiological properties of the female and male crane measured in this study. Changes for most input parameters are ±10% of values used in modeling Whooping Cranes, so that the sensitivity of model output to different parameters can be compared. The temperature range -25°C to 35°C was chosen to match the range of air temperatures that migratory Whooping Cranes experience in the wild^a^ and thus represent sensitivity analyses most relevant to modeling of real Whooping Cranes. Unless otherwise noted, metabolic-chamber-like conditions include low wind speed (0.01 m/s), low relative humidity (5%), no solar radiation, and ambient air temperatures changing in 1°C intervals. Solar radiation was set to 334.1W to analyze effects of changing solar reflectivity. For these analyses, all thermoregulation mechanisms (e.g. ptiloerection, vasodilation/constriction, panting) were turned off, so that the parameter value being tested would be the only value changing with air temperature. For reference, 1W is 8% of the basal metabolic rate for the simulated female crane and 7% of the basal metabolic rate for a simulated male crane. When metabolic rates drop to 0W within the range of temperatures modeled (S8–S10 Figs), only points with metabolic rates greater than 0 were included in the averages.

^a^The range of temperatures experienced by wild Whooping Cranes was approximated based on the range of air temperatures occurring at breeding grounds and wintering grounds of the two existing migratory populations of Whooping Cranes, the Eastern Migratory Population and the Aransas-Wood Buffalo Population during times of year when Whooping Cranes are at each location.

^b^Maximum value for reptile skin in lab database.

^c^ Minimum value for reptile skin in lab’s database.

^d^ Minimum value allowed in model for thermoregulation (See Table 2 for source).

^e^Maximum value allowed in model for thermoregulation (See Table 2 for source).

^f^For legs entire submerged in water of temperature equal to air temperature. At air temperatures of 0°C and lower, water temperature was held at 0.1°C.

The model was also somewhat sensitive to changes in whole-body solar reflectivity, with higher reflectivities leading to higher metabolic rates due to decreases in solar heat absorption. Average changes in modeled metabolic rate across the wide range of leg solar reflectivities modeled were only slightly larger than changes of ±10% for whole-body reflectivity, with the magnitude of leg reflectivity variation offsetting the smaller surface area of the legs.

Among other feather properties, changes of ±10% made a difference of <10% of basal metabolic rate. The model was more sensitive to changes in feather element diameter and feather layer depth and less sensitive to changes in density and length.

Modeled metabolic rates increased with feather element diameter. This increase occurred because feather elements have a greater conductivity than the still air between them. Effects were particularly pronounced at +50% of feather element diameter (S8 Fig). When this diameter was modeled, the feather elements were so closely packed that the model had to reduce element density values in order to incorporate the feather diameter. Modeled metabolic rates also increased with increasing feather density and length, though to a lesser extent.

Modeled metabolic rates decreased with increasing feather layer depth, as expected for increasing thickness of the insulating layer. Increasing thickness of the feather depth layer without increasing density/length/diameter of feather elements led to increasing volume of insulating columns of air.

As expected, all changes in feather properties (aside from solar reflectivity) had the greatest effect at colder temperatures, when the difference between skin and air temperature was larger (S8 Fig).

Subcutaneous fat content had little effect on modeled metabolic rates for cranes, from minimal fat (1%) to high fat contents similar to those measured in Sandhill Cranes at staging areas during spring migration [40] (S8 Fig).

The effect of changing core temperature from the measured value for Whooping Cranes to the minimum and maximum values allowed in the model for thermoregulation led to changes comparable to changing solar reflectivity by ±10%. Changes in the modeled minimum difference between average leg temperature and air/water temperature had the potential to make much larger changes in metabolic rate, although the differences disappear below the minimum boundary on leg temperature (S9 Fig).

## Discussion

### Model Performance

The similarity of Niche Mapper-simulated energy expenditures to doubly-labeled water-measured energy expenditures suggests that Niche Mapper accurately modeled Whooping Crane energy expenditure. The similarity of Niche Mapper-modeled energy expenditures to doubly-labeled water-measured energy expenditures occurred because doubly-labeled water-measured values were similar to values estimated based on activity energy alone (i.e. time-energy budgets) and Niche Mapper predicted no energy costs of thermoregulation for Whooping Cranes. Thus, Niche Mapper was able to independently simulate the daytime activity and nighttime resting metabolic rates estimated by the time-energy budget analysis without predicting thermoregulatory costs over the varying solar radiation levels, infrared radiation levels, wind speeds, air temperatures, and water temperatures experienced by the cranes.

The slightly higher doubly-labeled water-measured energy expenditure for the male could imply small thermoregulatory costs or a small level of inaccuracy in the time-energy budget (based on daytime behaviors or assumption of rest during nighttime). The slightly lower doubly-labeled water-measured energy expenditure for the female implies small inaccuracy in the model, as the cost of energy expenditure for activity alone cannot be less than total energy expenditure. In either case, it is also possible that error in the doubly-labeled water method (measurement error in isotope ratios or inaccurate assumptions in converting isotope loss rates to energy expenditures) could have contributed to the difference in values between doubly-labeled water-measured energy expenditures and modeled energy expenditures. In general, the female’s smaller energy expenditures were expected based on smaller body size.

The implied lack of thermoregulatory costs is not unreasonable given that the air temperature range experienced by the cranes, 0.5–27.6°C, fell well within the temperatures that Whooping Cranes experience in the wild, approximately -25°C to 35°C. (See S3 Text for how this temperature range was estimated.) The low energy-cost thermoregulatory responses simulated indicate that Niche Mapper was responsive to fluctuating environmental conditions. Further, the lack of simulated panting corresponded with visual observations. The model also performed well when tested against infrared-photograph measured leg temperatures, especially given that some variation existed even among infrared photograph-measured average leg temperatures within individual photographs (Fig 2). However, other thermoregulatory responses would not have been visible to observers, and it is not possible to partition thermoregulatory costs from total energy expenditure measured via doubly-labeled water to test for lack of thermoregulatory costs directly.

The model did overestimate temperatures of three legs at the highest air temperatures during which photographs were taken. The larger modeled-measured temperature difference may have occurred for multiple reasons. It is possible that the model over-estimated the increase in leg temperature needed for cranes to cool off under the combination of air temperatures, wind speeds, solar radiation, and long-wave infrared radiation experienced by cranes during the warmest periods of the day. It is also possible that the real cranes were engaging in other thermoregulatory mechanisms that allowed them to maintain their body temperature without increasing their leg temperatures further. In particular, the model engages in different types of thermoregulatory options one at a time when an animal is under thermal stress, proceeding from increasing thermal conductivity to maximum values, to increasing appendage temperatures to maximum values, to allowing slight increases in core temperature, to panting. It is possible that real cranes were engaging in small increases in core temperature or panting in conjunction with increasing leg temperatures, allowing them to increase heat loss to the environment without increasing leg temperatures to modeled values. We did not expect large birds to require substantial core temperature increases under the simulated microclimate conditions, but changes in core temperature with changing ambient temperatures have not been studied in Whooping Cranes and may occur. The similarity of energy expenditures calculated using time-energy budgets and doubly-labeled water measurements suggests that the cranes did not engage in energy-expensive thermoregulatory activities like panting. However, small amounts of energy expended on panting during short periods of time during the hottest part of the day may have gone undetected in this study.

Sensitivity analyses showed that the model is most sensitive to biological and environmental variables that were measured directly on the study animals and at the study site. The model’s sensitivity to morphometric dimensions demonstrates the importance of correcting morphometric measurements to body mass (via density) to obtain realistic surface areas when morphometric measurements may contain error, as was done in this study. It also demonstrates the importance of modeling potential variation in size among individuals in wild populations of Whooping Cranes (and other modeled animals). The model was also sensitive to changes in whole-body solar reflectivity. We are confident in the accuracy of our values for feather reflectivity because they were measured directly on Whooping Crane feathers, and values were similar to lab measurements (Porter WP, unpublished) of white feather solar reflectivities for other species, including Adelie Penguins (*Pygoscelis adeliae*), White Magpies (*Pica pica hudsonia*), Kingfisher (*Megaceryle alcyon alcyon*), and Red-headed Woodpeckers (*Melanerpes erythrocephalus*), which, except for one outlying value, all fell between 60 and 70%.

Changes of ±10% in biological variables not measured on our study animals resulted in only small changes (<10% of basal metabolic rate) in metabolic rate. The relatively small changes in feather element density and diameter across intermediate values are similar to the results of Porter *et al*. [51], which found little change in metabolic rates across a wide range of intermediate hair densities. The larger changes in metabolic rate that occurred at very large feather element diameters were also similar to the pattern found by Porter *et al*. [51]. In analyses across a wide range of fur densities, Porter *et al*. [51] found that very high hair densities led to a steep rise in metabolic rates because the hairs were so densely packed that they became effectively a solid insulation. However, Whooping Crane feather elements are not likely to be so large that their insulation is effectively solid, given that they are a species adapted to live in temperate regions. Further, values used to model Whooping Cranes also fell within the lower end of the range in feather barb diameters measured in a related species, the Siberian Crane (*Grus leucogeranus*), in Ilyashenko and Chernova [57]. Given that our values include both barbs and barbules, they are reasonable compared to measured values in Siberian Cranes.

One limitation of this study is that a sample size of only two individuals could be obtained for doubly-labeled water measurements. Therefore, we compared our measured energy expenditures to other measured values for Whooping Cranes to check for unreasonable values. Because the cranes in the study apparently experienced minimal energy costs of thermoregulation, total energy expenditure can be compared to values measured for captive Whooping Cranes using indigestible markers in food [23] (n = 5 individuals). Temperatures experienced by Whooping Cranes during the study period were unlikely to lie outside the thermoneutral zone [23] and thus probably do not include thermoregulatory costs. Daily energy expenditure values in Nelson [23] ranged from 1.7–2.0x BMR (using Equation 3 in McNab [42] to estimate basal metabolic rate given the body masses reported in Nelson [23]), with an outlying value of 2.4xBMR. The measured and Niche Mapper-modeled daily energy expenditures (and energy expenditure estimates based on time-energy budgets) are similar, falling within the range of 1.7 to 2.0xBMR (Tables 1 and 5). The similarity of our measured values to those of Nelson [23] does not provide direct evidence of the accuracy of our doubly-labeled measurements. However, given that captive Whooping Cranes are expected to have similar behaviors (activity energy expenditures) across studies values measured in Nelson [23] do not raise concerns about unreasonable values in our doubly-labeled water measurements.

Another limitation of this study is that the captive cranes never experienced thermoregulatory energy costs, so that the model’s ability to accurately simulate these costs at more extreme temperatures remains untested. Energy expenditures of this endangered species have never been measured outside of (assumed) thermoneutral conditions, and are thus not available from the literature, either.

For reference, the energy expenditures of Whooping Cranes in metabolic chamber-like conditions over a wider range of air temperatures were simulated and compared to allometric predictions. Niche Mapper predicted a lower critical temperature of 1°C for Whooping Cranes. An allometric equation for lower critical temperatures of non-passerine birds [50, Eqn. 20] (based on allometric regressions of basal metabolic rate and heat transfer coefficients) predicts values of -8°C for the female crane and -11°C for the male crane, which differ considerably from the 1°C predicted by Niche Mapper. However, Calder and King [50] point out that lower critical temperature can vary widely for species of a given size. For example, allometric equations predict a lower critical temperature of -17°C for mute swans (mean body size = 8.3 kg; [58]), but Bech [58] empirically measured a lower critical temperature of 1°C for mute swans. Therefore, the fact that Niche Mapper's lower critical temperature prediction is approximately 10°C greater than predicted by allometric equations does not necessarily mean that Niche Mapper cannot accurately predict the lower critical temperatures of cranes. Direct testing on live animals would be needed to clarify the issue.

Below lower critical temperature, values of minimal conductance (heat loss divided by the difference between deep body temperature and ambient temperature, when ambient temperatures lie below the thermoneutral zone; [56]) can be compared to values predicted by allometric equations in Aschoff [56]. For non-passerine birds measured during the active phase, the allometric equation predicts values of 0.42 W/°C for the female crane and 0.46 W/°C for the male crane. Model values were similar, with an average of 0.51 W/°C for the female crane and 0.57 W/°C for the male crane over modeled air temperatures of -25 to 0°C, the range of temperatures below the thermoneutral zone that Whooping Cranes are likely to experience in the wild. The slightly larger value for the male crane, despite its larger size, is likely due to small morphometric differences from the female.

### Implications

Our study was the first to examine how Whooping Crane leg temperatures vary with environmental temperatures. We found that Whooping Cranes can reduce leg temperature in response to low air temperatures, and that leg temperatures can be reduced at least as low as approximately 4°C. This is near minimal temperatures that animals must maintain to keep tissues from freezing. This information will be important in considering heat loss and environmental tolerances of wild Whooping Cranes. In particular, future modeling of Whooping Cranes will more accurately reflect their ability to withstand cold temperatures and improve simulation of energy requirements necessary to thermoregulate in early spring and in northern latitudes.

This mechanistic model will provide an accurate, non- invasive way to estimate the energy expenditure of endangered wild Whooping Cranes. Energy expenditure on activity can be estimated from time-energy budgets of wild cranes, and biological variation in allometry, etc., across individuals can be estimated from literature values. Our results show that Niche Mapper, as informed by a time-energy budget, accurately models the energy expenditure of Whooping Cranes across a variety of environmental conditions compared to the doubly-labeled water method.

The capabilities of this mechanistic model, with additions allowing for wading behavior and counter-current heat exchange, can now be used to address questions on broad time and geographic scales for this wide-ranging species and to inform conservation decisions. For example, Niche Mapper’s ability to model endotherm energetics across landscapes based on user-input rasters of climate and topographic data will allow future studies to address questions about effects of local climate, migration timing, and food availability on the energy expenditure, and associated health, habitat requirements, and breeding success, of Whooping Cranes in different populations. This information can be used to assess current and potential future reintroduction locations, and to tie conditions on wintering locations and breeding locations together. In particular, a mechanistic understanding of the fundamental niche of Whooping Cranes may reveal potential range beyond that revealed by correlations based on current realized niche conditions. Possible direct effects of climate change on Whooping Crane energy expenditure, temperature regulation, and behavior can also be explored. Individual input variables can be varied to find what aspects of environment and behavior are most influential for Whooping Cranes, and to assess possible future reintroduction locations.

To our knowledge, this is also the first study to mechanistically model the energy expenditure a bird using counter-current heat exchange in its appendages. Simulations of Whooping Cranes in metabolic-chamber like conditions suggest that reducing average leg temperature to near environmental conditions makes a large difference in heat loss (and energy expenditure) of long-legged birds, especially when wading. Simulated metabolic rates increased by up to 180% for non-wading cranes and by up to 750% for wading cranes when counter-current heat exchange in legs was removed from simulations. While these simulations remain to be tested against measured values, the large increase in simulated metabolic rates combined with the accuracy of our simulated daily energy expenditures compared to doubly-labeled water results suggest that counter-current exchange was indeed important to include in modeling wading cranes. Given the potential for large changes in simulated energy expenditure with the inclusion of counter-current exchange, our additions to Niche Mapper will improve the accuracy of ecological energetics modeling for not only Whooping Cranes, but also other birds and mammals that use counter-current heat exchange in their appendages. Our model can also now be used to examine the importance of counter-current exchange to the heat balance and environmental tolerances of different species, given varying leg dimensions and the species-specific patterns of blood vessels allowing for varying responses to changes in ambient temperatures. Analyses could be carried out across a range of appendage parameter values, or Niche Mapper could be parameterized using measured appendage temperatures from a variety of species, as we have done for Whooping Cranes. This type of analysis could give particular insight into the importance of counter-current exchange systems for species that must engage in behaviors that tend to increase heat loss, such as wading and diving, in order to forage or travel.

Our addition of wading behavior to Niche Mapper will also allow for more accurate modeling of species that wade. While Niche Mapper previously had the capability to model diving animals, it could not simulate the effects of having legs alone submerged at varying depths for varying parts of the day. Niche Mapper can now be used to evaluate the effects of varying amounts of time and times of day spent in water, given water temperatures in different locations. The ability to wade in hot temperatures may expand the thermal tolerances and associated ranges of some species. Conversely, a need to wade (to obtain food, for example) could reduce thermal tolerances or range in cooler climates. Having this simulation capability in a model will allow future researchers to examine such questions across large geographic areas, potentially allowing for exploration of species range limits as they relate to thermal tolerances, wading behavior, and the evolution of counter-current exchange mechanisms.

## Supporting Information

S1 Fig Hourly average air temperatures measured outside Whooping Crane enclosure during doubly-labeled water measurement period at four heights above ground (2m, 1m, 0.5m, and 0.1 m). Air temperatures were measured once per minute using shaded thermocouples, and 15-minute averages were recorded by a datalogger.
(TIF)

S2 Fig Average water temperatures measured in the wading pond in the Whooping Crane enclosure during doubly-labeled water measurement period at three depths (1–2 cm below surface, 30 cm below surface, and 60cm below surface) at 15-minute intervals. Water temperatures were measured once per minute using thermocouples, and average values were recorded by a datalogger every 15 minutes. On 9/26, it was discovered that the thermocouple measuring water temperature at 1–2 cm depth had detached from its position and floated to the surface, leading to readings that closely followed air temperature. Because 1–2 cm depth readings closely followed 30cm depth temperatures on the first day of the experiment and after the thermocouple was repositioned (11:11AM 9/26), 1–2cm depth readings between 11:00AM on 9/25 and 11:11AM on 9/26 were considered erroneous based on divergence from 30cm depth temperatures. Temperatures at 30cm depth were used to estimate average water temperature along the length of the leg. Temperatures at 30cm depth were very close to water surface temperatures (average 0.18°C, range -0.34°C to 2.4°C), and Whooping Crane leg lengths were not much longer than 30cm (37.5 cm and 39.5cm). Further, the difference between 39.5cm water depth and 30cm water depth (assuming linear temperature change between 30cm and 60cm water depth temperature) was small (range -1.12°C to -0.24°C) and would have little effect on average water temperature along the length of the leg.
(TIF)

S3 Fig Average wind speeds measured in 15-minutes intervals outside the Whooping Crane enclosure during doubly-labeled water measurement period at four heights above ground (2m, 1m, 0.5m, and 0.1 m). Wind speeds were measured using 3-cup anemometers (Rimco) with total anemometer rotations recorded by a datalogger every 15 minutes.
(TIF)

S4 Fig Fifteen-minute averages of solar radiation (horizontal plane) measured outside Whooping Crane enclosure during doubly-labeled water measurement period at four heights above ground (2m, 1m, 0.5m, and 0.1 m). Solar radiation was measured once per minute using a Campbell Scientific CS300 pyranometer, with average values recorded by a data logger every 15 minutes.
(TIF)

S5 Fig Proportions of time spent in different behaviors by two Whooping Cranes (one adult male, one adult female) during daylight hours of a four-day period during which energy expenditure was measured using the doubly-labeled water technique. The proportion of time spent in social behavior was very small (0.2% for each individual).
(TIF)

S6 Fig Daily proportions of time spent in different behaviors by two captive Whooping Cranes (one adult male, one adult female) during daylight hours of a four-day period during which energy expenditure was measured using the doubly-labeled water technique. (TIF)

S7 Fig Proportions of time spent in different behaviors by two captive Whooping Cranes (one adult male, one adult female) during daylight hours of a four-day period during which energy expenditure was measured using the doubly-labeled water technique. Hour 6 incorporates all observations between 6:00 and 6:59 across all days, Hour 7 incorporates 7:00–7:59, etc.
(TIF)

S8 Fig Changes in modeled Whooping Crane metabolic rate in metabolic-chamber-like conditions in response to varying morphometric, physiological, and feather property input parameters for sensitivity analyses. Input parameters varied are (A) feather element diameter, (B) feather element density, (C) feather length, (D) feather layer depth, (E) body fat content, and (F) allometry (length and width of body parts). All original values can be found in Tables **2**and **3**. Unless otherwise noted, results are modeled using the morphology of the female crane in this study. Sensitivity analyses using the male crane’s morphology had similar results. Feather property variations were chosen to represent a wide range of values. Maximum body fat values were chosen based on values measured for Sandhill Cranes at staging areas during spring migration in [40]. Morphometric values were varied by ±10%. Because final values used in the model were adjusted based on the density of birds, the adjusted values are also shown.
(TIF)

S9 Fig Changes in modeled Whooping Crane metabolic rate in metabolic-chamber-like conditions in response to varying solar reflectivity and body temperature input parameters for sensitivity analyses. Input parameters varied are (A) solar reflectivity, (B) leg reflectivity, (C) core temperature, (D) minimum difference between leg core temperature and air temperature, and (E) minimum difference between leg core temperature and water temperature for a crane with legs entirely submerged in water. Original values can be found in Tables **2**and **3**. Unless otherwise noted, results are modeled using the morphology of the female crane in this study. Sensitivity analyses using the male crane’s morphology had similar results. Minimum and maximum leg solar reflectivities are minimum and maximum values available for reptile skins in lab database. Body core tempeartuers are varied to the minimum and maximum values allowed in the model for thermoregulation.
(TIF)

S10 Fig Changes in modeled Whooping Crane metabolic rate in metabolic-chamber-like conditions in response to varying environmental input variables for sensitivity analyses. Input parameters varied are (A) wind speed and (B) solar radiation. Wind speeds ranging from 0m/s to average daily maximum measured during doubly-labeled water measurements are shown. Solar radiation values ranging from 0W to the average daily maximum are shown.
(TIF)

S1 Table Times during which the behaviors of a two captive Whooping Cranes (one adult male, one adult female) were video-recorded and sunrise/sunset times over approximately four days in September 2012. (DOCX)

S2 Table Average energy expenditure on activity during daylight hours for two Whooping Cranes (one male, one female) over a four-day period during which energy expenditure was measured using the doubly-labeled water technique, based on time budgets summarized using two alternate methods. (DOCX)

S3 Table Concentrations of deuterium (D) and ^18^O in blood plasma of two captive Whooping Cranes (one male and one female) prior to (“background”) and following injection of doubly-labeled water (D_2_^18^O). (DOCX)

S1 Text Rationale for use of food pellet composition in determining respiratory quotient to interpret doubly-labeled water measurements for two captive Whooping Cranes. (DOCX)

S2 Text Time-activity-energy budgets of captive Whooping Cranes. (DOCX)

S3 Text Estimation of air temperatures experienced by wild migratory Whooping Cranes. (DOCX)
