# Supplementary figures and images for: Validation of a Mechanistic Model for Non-Invasive Study of Ecological Energetics in an Endangered Wading Bird with Counter-Current Heat Exchange in its Legs
Source: PLoS One. 2015 Aug 26;10(8):e0136677. doi: 10.1371/journal.pone.0136677 (PMC4550283; doi:10.1371/journal.pone.0136677)

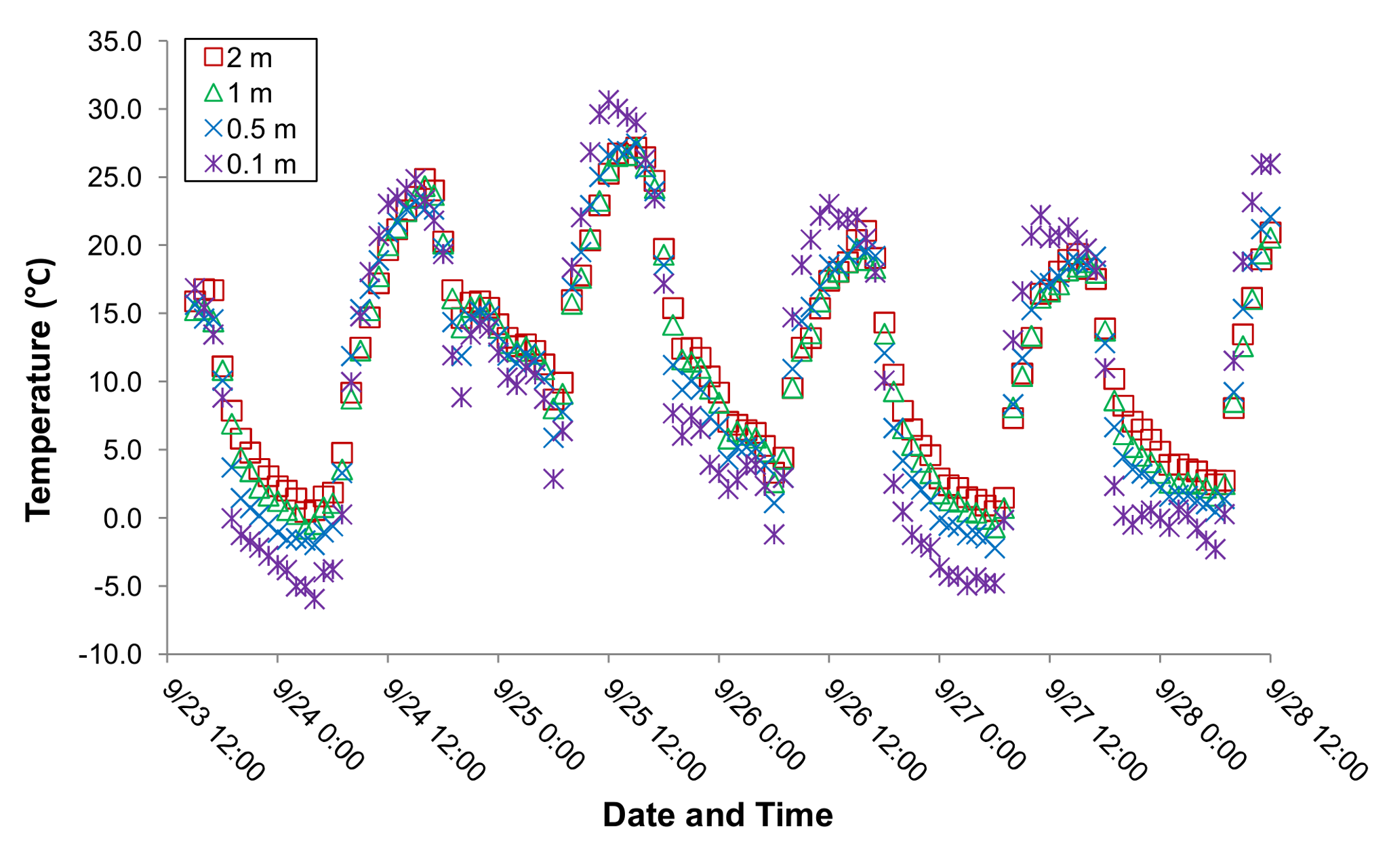

Supplement: S1 Fig — Air temperatures were measured once per minute using shaded thermocouples, and 15-minute averages were recorded by a datalogger. (TIF) [file pone.0136677.s001.tif]

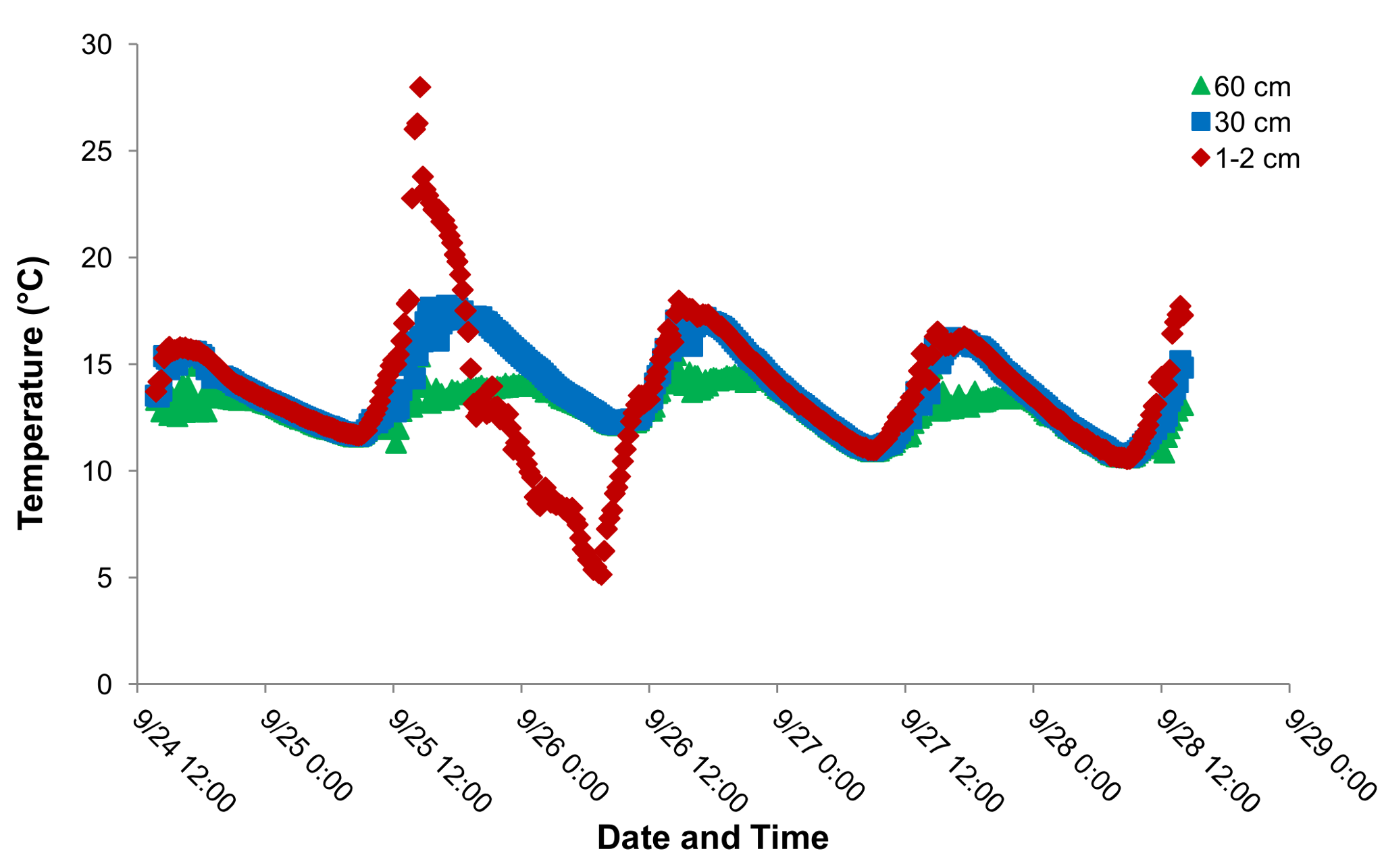

Supplement: S2 Fig — Water temperatures were measured once per minute using thermocouples, and average values were recorded by a datalogger every 15 minutes. On 9/26, it was discovered that the thermocouple measuring water temperature at 1–2 cm depth had detached from its position and floated to the surface, leading to readings that closely followed air temperature. Because 1–2 cm depth readings closely followed 30cm depth temperatures on the first day of the experiment and after the thermocouple was repositioned (11:11AM 9/26), 1–2cm depth readings between 11:00AM on 9/25 and 11:11AM on 9/26 were considered erroneous based on divergence from 30cm depth temperatures. Temperatures at 30cm depth were used to estimate average water temperature along the length of the leg. Temperatures at 30cm depth were very close to water surface temperatures (average 0.18°C, range -0.34°C to 2.4°C), and Whooping Crane leg lengths were not much longer than 30cm (37.5 cm and 39.5cm). Further, the difference between 39.5cm water depth and 30cm water depth (assuming linear temperature change between 30cm and 60cm water depth temperature) was small (range -1.12°C to -0.24°C) and would have little effect on average water temperature along the length of the leg. (TIF) [file pone.0136677.s002.tif]

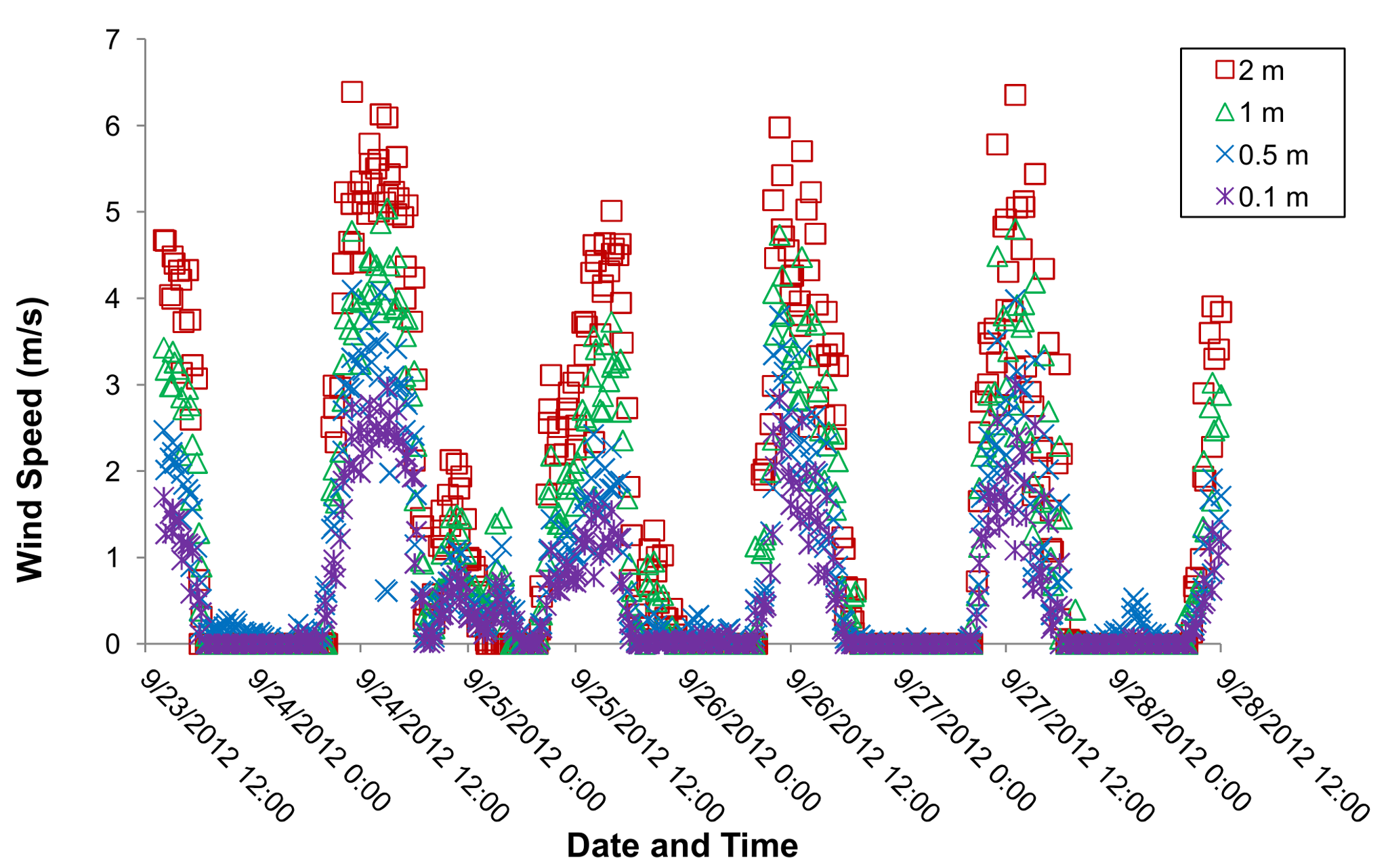

Supplement: S3 Fig — Wind speeds were measured using 3-cup anemometers (Rimco) with total anemometer rotations recorded by a datalogger every 15 minutes. (TIF) [file pone.0136677.s003.tif]

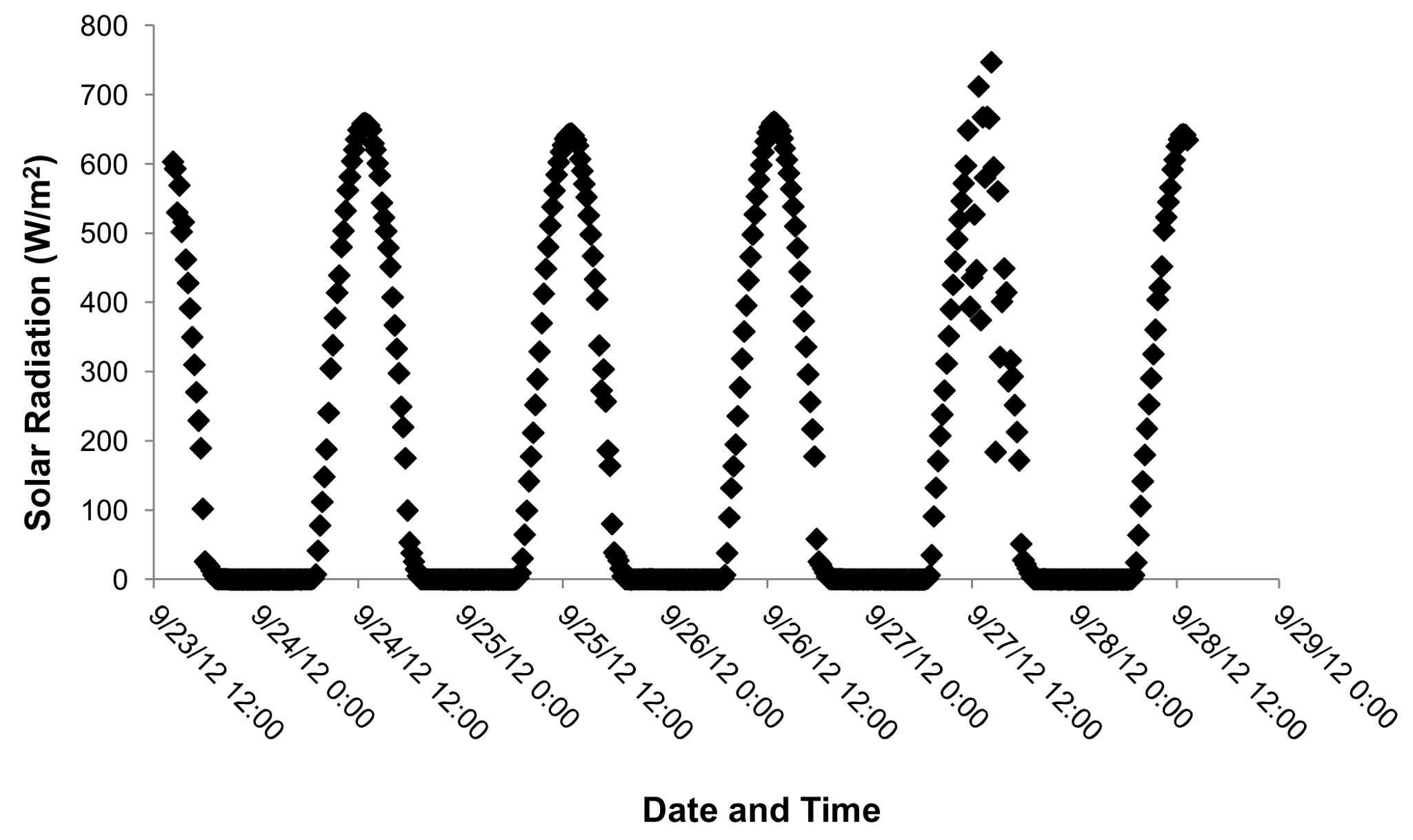

Supplement: S4 Fig — Solar radiation was measured once per minute using a Campbell Scientific CS300 pyranometer, with average values recorded by a data logger every 15 minutes. (TIF) [file pone.0136677.s004.tif]

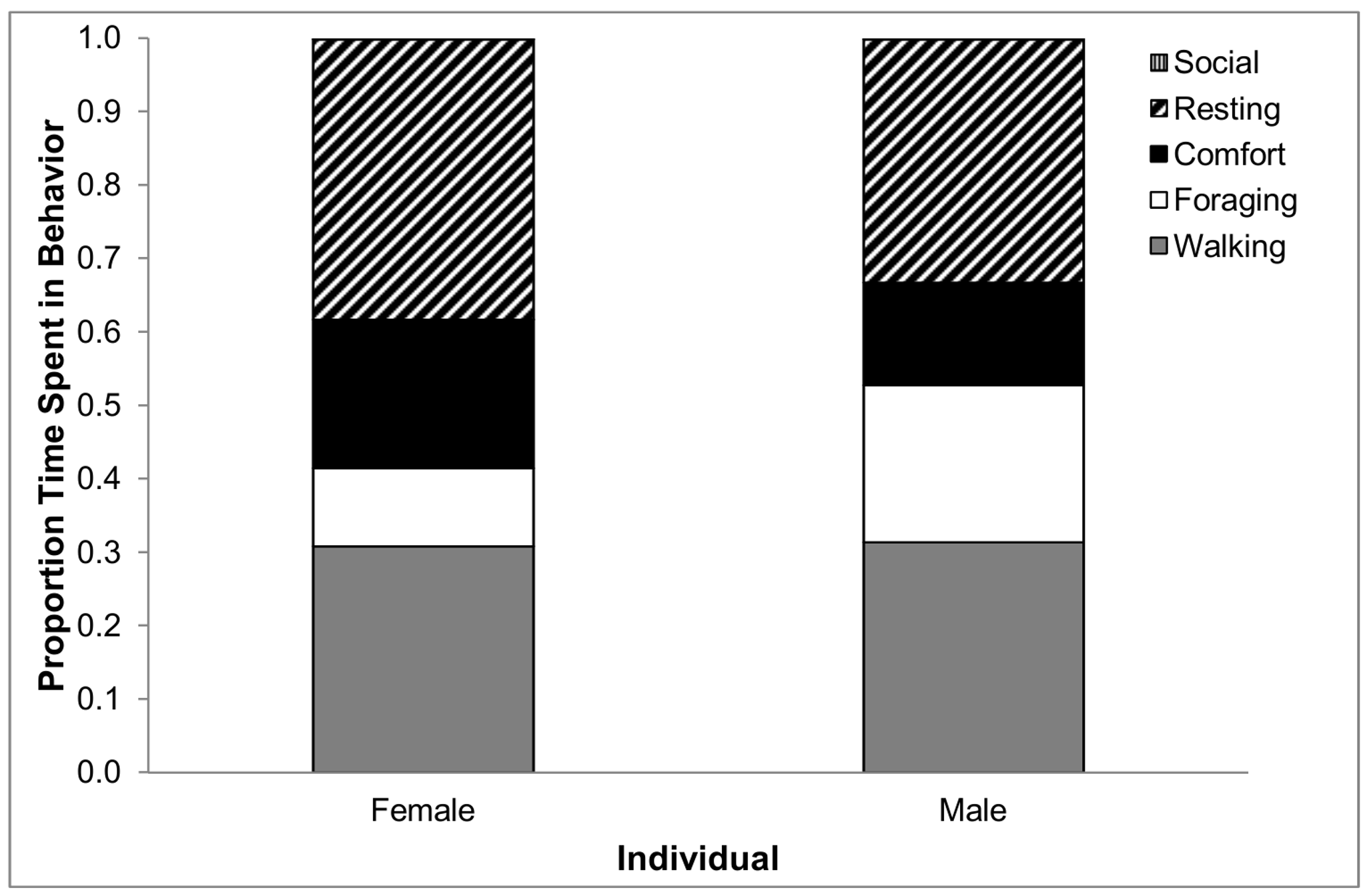

Supplement: S5 Fig — The proportion of time spent in social behavior was very small (0.2% for each individual). (TIF) [file pone.0136677.s005.tif]

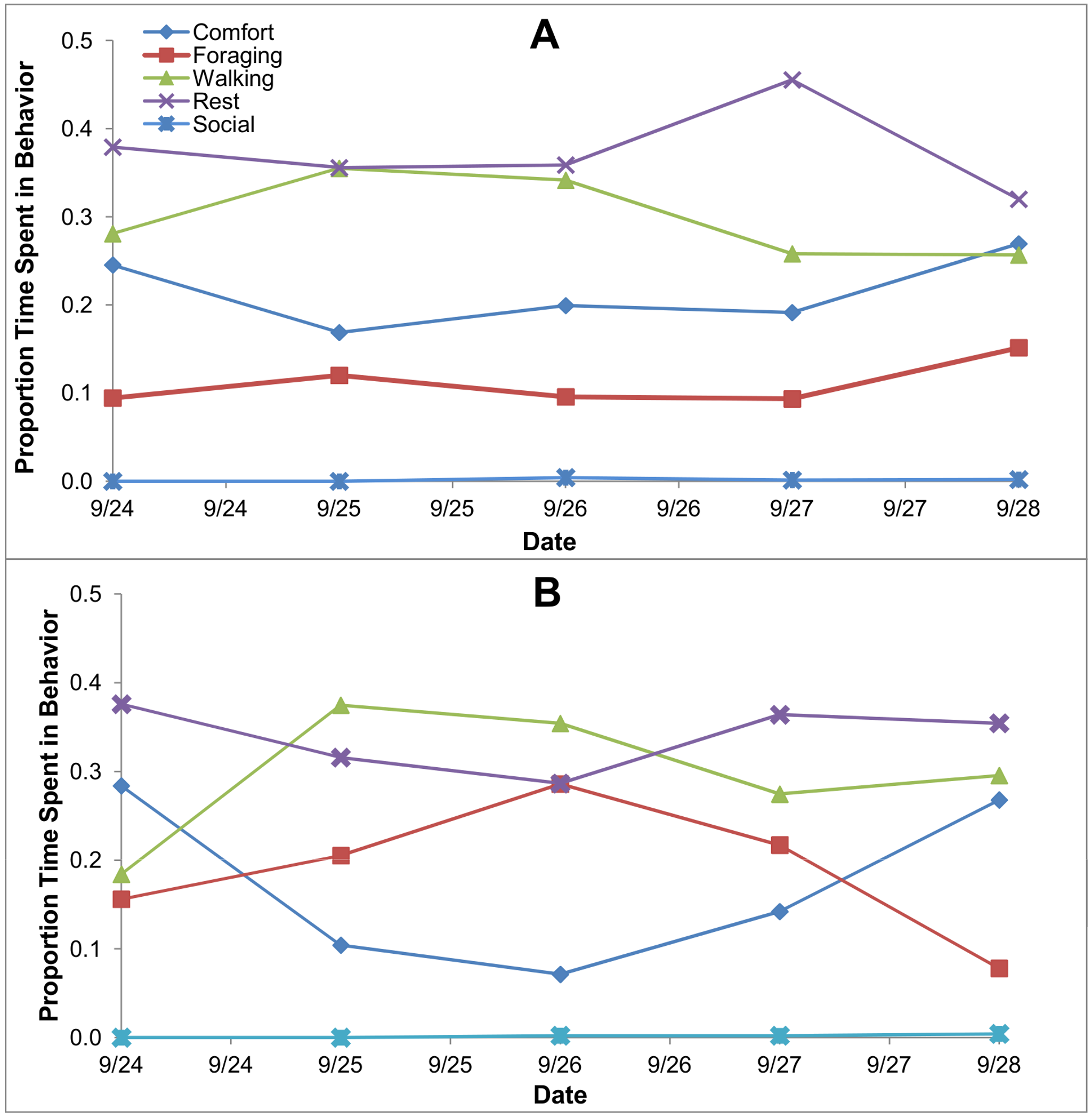

Supplement: S6 Fig — (TIF) [file pone.0136677.s006.tif]

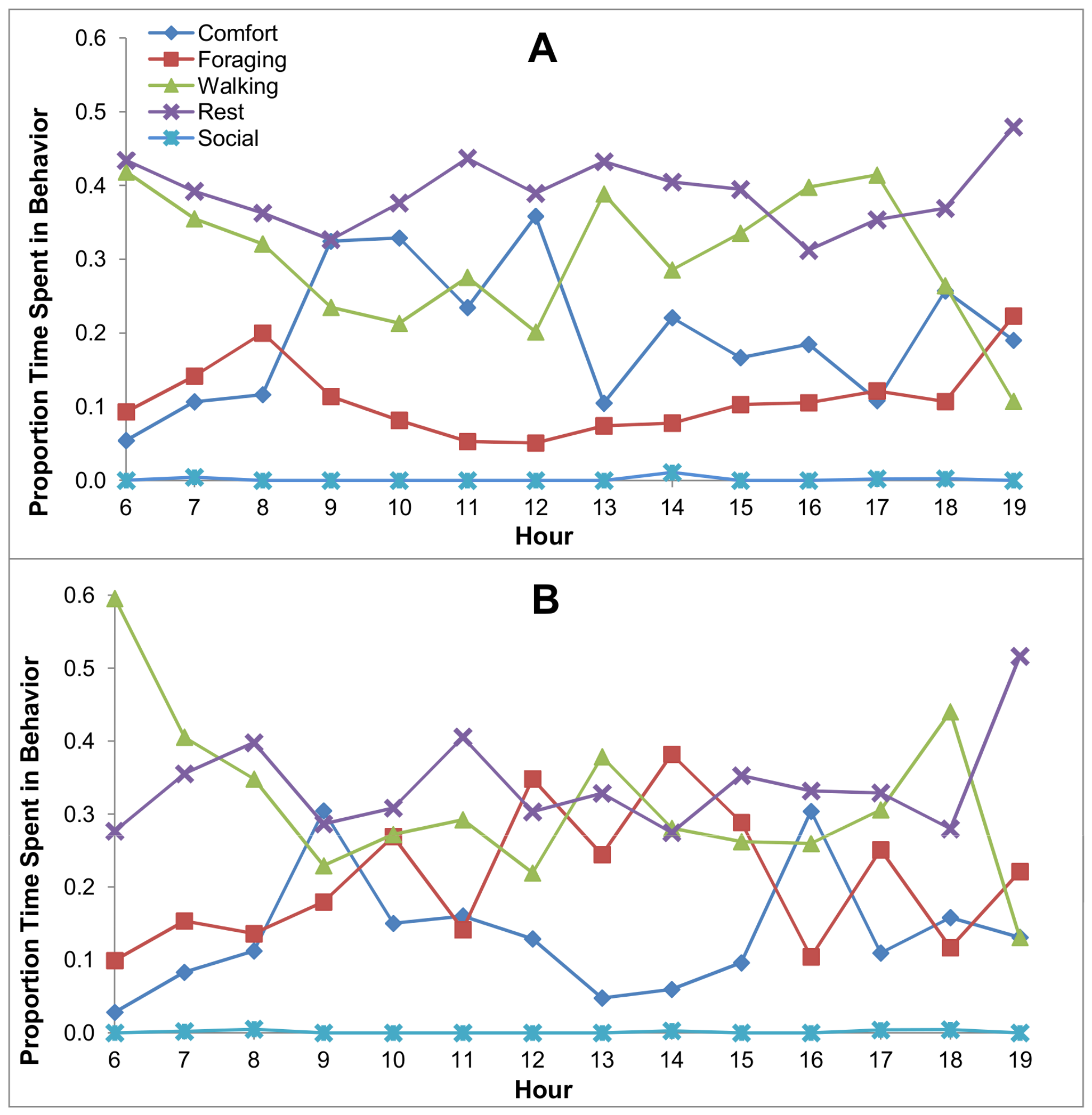

Supplement: S7 Fig — Hour 6 incorporates all observations between 6:00 and 6:59 across all days, Hour 7 incorporates 7:00–7:59, etc. (TIF) [file pone.0136677.s007.tif]

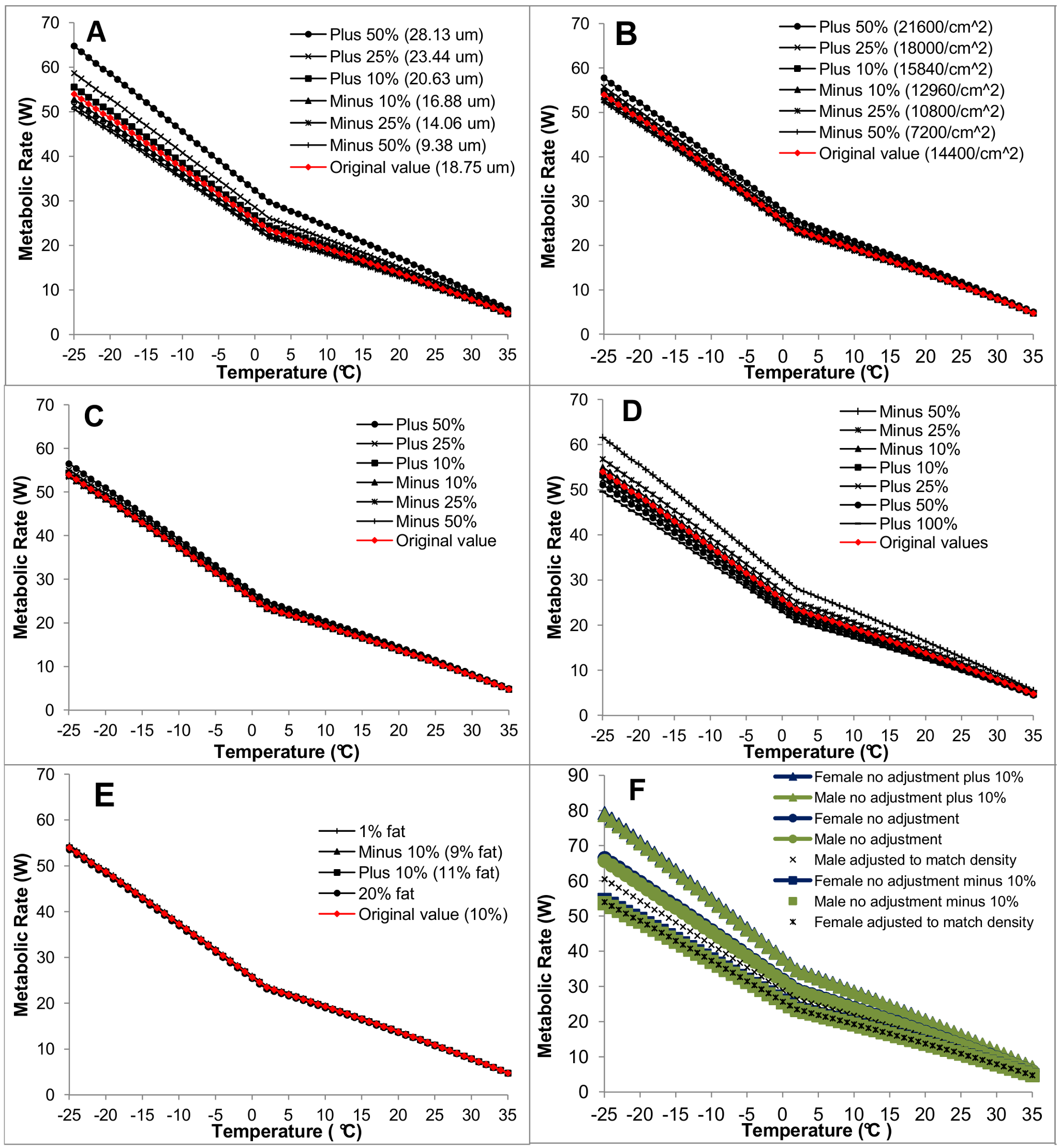

Supplement: S8 Fig — Input parameters varied are (A) feather element diameter, (B) feather element density, (C) feather length, (D) feather layer depth, (E) body fat content, and (F) allometry (length and width of body parts). All original values can be found in Tables 2and 3. Unless otherwise noted, results are modeled using the morphology of the female crane in this study. Sensitivity analyses using the male crane’s morphology had similar results. Feather property variations were chosen to represent a wide range of values. Maximum body fat values were chosen based on values measured for Sandhill Cranes at staging areas during spring migration in [40]. Morphometric values were varied by ±10%. Because final values used in the model were adjusted based on the density of birds, the adjusted values are also shown. (TIF) [file pone.0136677.s008.tif]

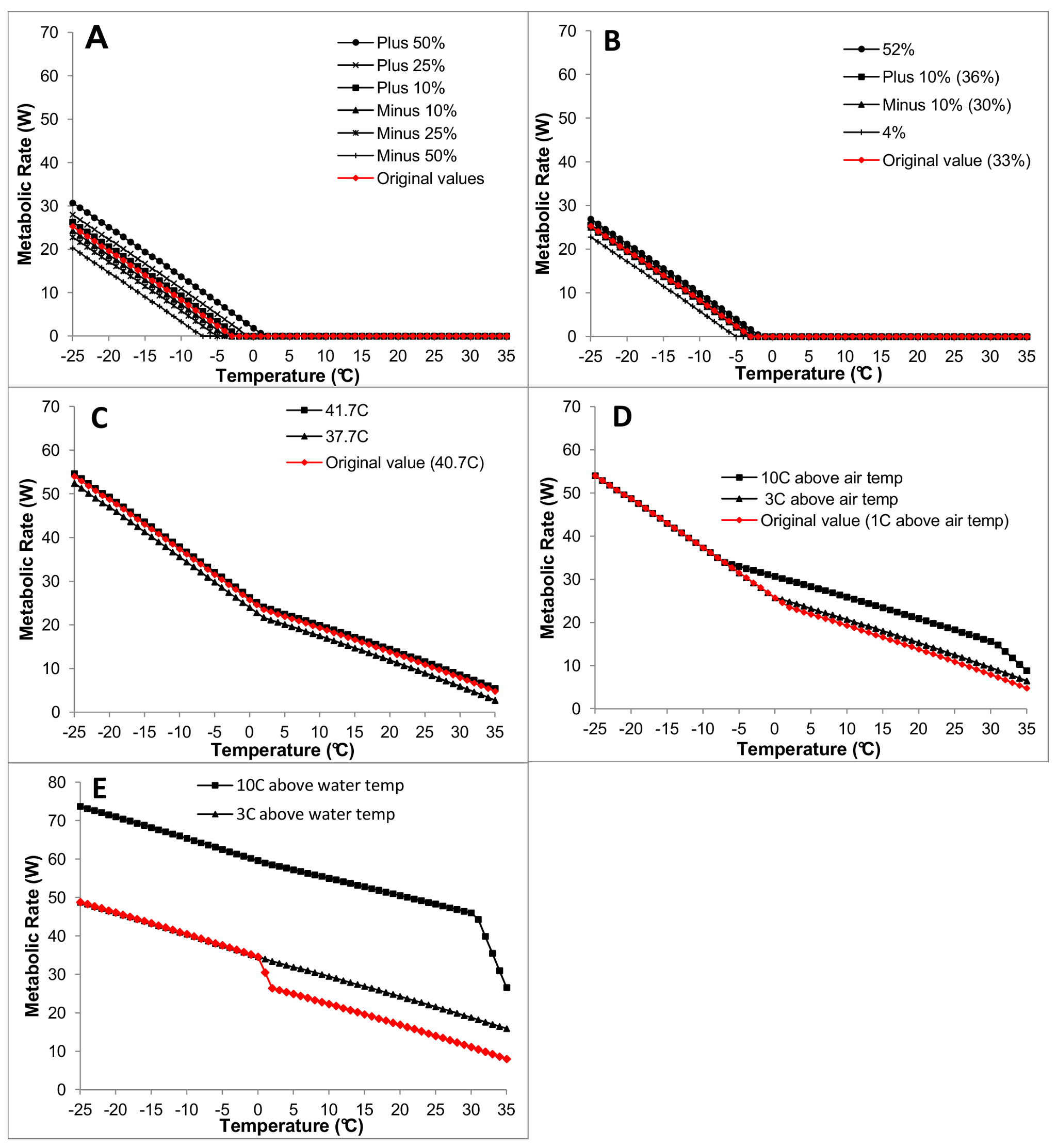

Supplement: S9 Fig — Input parameters varied are (A) solar reflectivity, (B) leg reflectivity, (C) core temperature, (D) minimum difference between leg core temperature and air temperature, and (E) minimum difference between leg core temperature and water temperature for a crane with legs entirely submerged in water. Original values can be found in Tables 2and 3. Unless otherwise noted, results are modeled using the morphology of the female crane in this study. Sensitivity analyses using the male crane’s morphology had similar results. Minimum and maximum leg solar reflectivities are minimum and maximum values available for reptile skins in lab database. Body core tempeartuers are varied to the minimum and maximum values allowed in the model for thermoregulation. (TIF) [file pone.0136677.s009.tif]

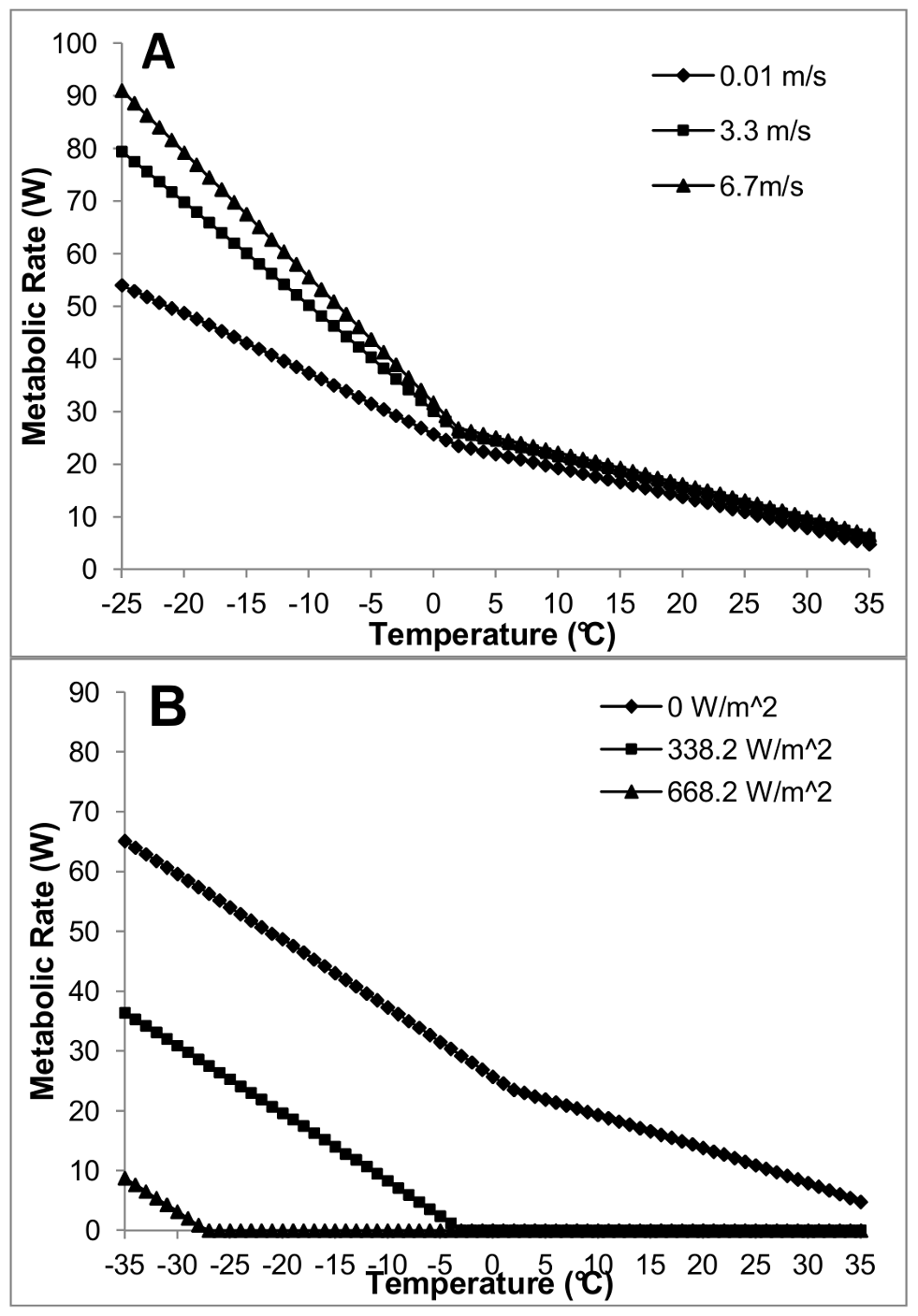

Supplement: S10 Fig — Input parameters varied are (A) wind speed and (B) solar radiation. Wind speeds ranging from 0m/s to average daily maximum measured during doubly-labeled water measurements are shown. Solar radiation values ranging from 0W to the average daily maximum are shown. (TIF) [file pone.0136677.s010.tif]
