# Supplementary material for: Validation of a Mechanistic Model for Non-Invasive Study of Ecological Energetics in an Endangered Wading Bird with Counter-Current Heat Exchange in its Legs
Source: PLoS One. 2015 Aug 26;10(8):e0136677. doi: 10.1371/journal.pone.0136677 (PMC4550283; doi:10.1371/journal.pone.0136677)
Supplement: S1 Table — (DOCX) [file pone.0136677.s011.docx]

Table S1. Times during which the behaviors of a two captive Whooping Cranes (one adult male, one adult female) were video-recorded and sunrise/sunset times over approximately four days in September 2012.

| Individual | Date | Time start | Time stop | Sunrise^a^ | Sunset^a^ |
| --- | --- | --- | --- | --- | --- |
| Female | 9/24/2012 | 12:05:30 | 19:15:00 | 6:49 | 18:52 |
| Male |  | 12:05:30 | 19:16:00 |  |  |
| Female | 9/25/2012 | 6:49:00 | 19:14:30 | 6:50 | 18:50 |
| Male |  | 6:49:00 | 19:14:30 |  |  |
| Female | 9/26/2012 | 6:55:00 | 19:14:30 | 6:51 | 18:48 |
| Male |  | 6:55:00 | 19:14:00 |  |  |
| Female | 9/27/2012 | 6:40:30 | 19:15:00 | 6:52 | 18:46 |
| Male |  | 6:41:00 | 19:16:00 |  |  |
| Female | 9/28/2012 | 6:29:30 | 10:36:00 | 6:54 | 18:45 |
| Male |  | 6:24:30 | 10:35:00 |  |  |
| Start and stop times are given to the nearest 30-second interval.  ^a^Sunrise and sunset times for 43°32’52.58”N, 89°45’22.38”W from <aa.usno.navy.mil/data/docs/RS_OneDay.php>. | | | | | |
