# Supplementary material for: Validation of a Mechanistic Model for Non-Invasive Study of Ecological Energetics in an Endangered Wading Bird with Counter-Current Heat Exchange in its Legs
Source: PLoS One. 2015 Aug 26;10(8):e0136677. doi: 10.1371/journal.pone.0136677 (PMC4550283; doi:10.1371/journal.pone.0136677)
Supplement: S2 Table — (DOCX) [file pone.0136677.s012.docx]

Table S2. Average energy expenditure on activity during daylight hours for two captive Whooping Cranes (one male, one female) over a four-day period during which energy expenditure was measured using the doubly-labeled water technique, based on time budgets summarized using two alternate methods.

|  | Energy Expenditure (xBMR) using Method 1 | | Energy expenditure (xBMR) using Method 2 | |
| --- | --- | --- | --- | --- |
| Date | Female | Male | Female | Male |
| 9/24/2014 | 2.34 | 2.25 | 2.30 | 2.23 |
| 9/25/2014 | 2.47 | 2.51 | 2.42 | 2.46 |
| 9/26/2014 | 2.46 | 2.48 | 2.47 | 2.54 |
| 9/27/2014 | 2.33 | 2.36 | 2.35 | 2.38 |
| 9/28/2014 | 2.33 | 2.39 | 2.33 | 2.40 |
| Avg. | 2.39 | 2.39 | 2.37 | 2.39 |
| St.dev | 0.07 | 0.12 | 0.07 | 0.13 |
| Energy expenditures are reported as multiples of basal metabolic rate (BMR). In Method 1, time spent in a given activity was calculated across the whole day. In Method 2, hourly proportions of time spent in an activity were calculated, and values were averaged across hours. (See Text S2 for more details.) Method of calculating time budgets made little difference in activity multipliers. Values from Method 1 were used for model input. | | | | |
