# Supplementary material for: Validation of a Mechanistic Model for Non-Invasive Study of Ecological Energetics in an Endangered Wading Bird with Counter-Current Heat Exchange in its Legs
Source: PLoS One. 2015 Aug 26;10(8):e0136677. doi: 10.1371/journal.pone.0136677 (PMC4550283; doi:10.1371/journal.pone.0136677)
Supplement: S3 Table — (DOCX) [file pone.0136677.s013.docx]

S3 Table. Concentrations of deuterium (D) and ^18^O in blood plasma of two captive Whooping Cranes (one male and one female) prior to (“background”) and following injection of doubly-labeled water (D_2_^18^O).

|  | Male | Female |
| --- | --- | --- |
| Background D (δ) | -44.31 | -43.49 |
| Sample 1 D (δ) | 601.20 | 803.97 |
| Sample 2 D (δ) | 145.77 | 233.28 |
| Background ^18^O (δ) | -5.46 | -5.825 |
| kD (δ) | 0.308 | 0.283 |
| Sample 1 ^18^O (δ) | 80.345 | 107.525 |
| Sample 2 ^18^O (δ) | 15.095 | 24.53 |
| k^18^O (δ) | 0.360 | 0.333 |

Calculated values of daily decline in D and ^18^O (kD and k^18^O; [22]) are also shown. For each crane, 0.10 g H_2_^18^O (97 AP) kg^-1^ and 0.13 g D_2_O (99.9 AP) kg^-1^ were combined, diluted 5:2 with 3% physiological saline, and delivered via intravenous injection to the jugular vein. The time between the doubly-labeled water dose and first sample was 2 hours and 23 minutes for the male crane and 2 hours and 32 minutes for the female crane. The time between the first and second samples was 3 days, 23 hours, and 10 minutes for the male and 3 days, 23 hours, and 2 minutes for the female. Samples were spun to separate the plasma and passed through a 10kDa exclusionary filter (Vivaspin GE Healthcare) to remove the large protein portion prior to analysis. Deuterium was measured using a Thermo Scientific DeltaPlus isotope ratio mass spectrometer and ^18^O was measured using a Thermo Scientific Delta V.
