# Supplementary material for: Validation of a Mechanistic Model for Non-Invasive Study of Ecological Energetics in an Endangered Wading Bird with Counter-Current Heat Exchange in its Legs
Source: PLoS One. 2015 Aug 26;10(8):e0136677. doi: 10.1371/journal.pone.0136677 (PMC4550283; doi:10.1371/journal.pone.0136677)
Supplement: S1 Text — (DOCX) [file pone.0136677.s014.docx]

The two Whooping Cranes measured in this study consumed provided food (in pellet) and prey items occurring naturally in their enclosure, primarily crayfish (*Orconectes rusticus*) and bulrush tubers (*Scirpus acutus*). The composition of food pellets was used to interpret doubly-labeled water results because the composition of non-pellet food items made little difference in the final energy expenditure calculations. This was determined as follows. The range of possible daily energy expenditures for each crane was calculated from measured CO_2_ expiration rates by assuming a pure protein diet, a pure lipid diet, and then a pure carbohydrate diet. Total energy that cranes obtained from pellets was estimated on the basis of food composition and a metabolizable energy coefficient of 0.693 (measured for Whooping Cranes consuming crane breeder feed, which is similar in composition to food pellets in our study, in [1]). If each crane ate half of the food consumed from the bucket, energy from pellets accounted for, at minimum (assuming a lipid diet, which is unlikely given the low lipid content of crayfish and bulrush tubers), 64% of the daily energy expenditure of a crane. We then calculated the range of possible energy expenditures given a diet that was 64% pellets and 36% pure protein, pure carbohydrate, or pure lipid, and compared them to energy expenditure calculated for a diet of 100% pellets. If cranes ate 100% protein or carbohydrates for the non-pellet portions of their diet, calculated energy expenditures were within 5% of energy expenditures calculated under pure pellet diet (-1.8% for carbohydrates and 5.0% for lipids). If the remainder of the crane diet consisted of lipids, energy expenditure calculations increased by 10.4%, but this is unlikely given the low lipid content of crayfish and bulrush tubers.

**References**

1. Nelson JT (1995) Nutritional quality and digestibility of foods eaten by Whooping Cranes on their Texas wintering grounds. M.Sc. Thesis, Texas A&M University.
