# Supplementary material for: Validation of a Mechanistic Model for Non-Invasive Study of Ecological Energetics in an Endangered Wading Bird with Counter-Current Heat Exchange in its Legs
Source: PLoS One. 2015 Aug 26;10(8):e0136677. doi: 10.1371/journal.pone.0136677 (PMC4550283; doi:10.1371/journal.pone.0136677)
Supplement: S2 Text — (DOCX) [file pone.0136677.s015.docx]

One important input into the Niche Mapper for modeling energy expenditure is the daily energy cost of activities (e.g. walking, flying, etc.) for each day being modeled. This value is input as a multiple of basal metabolic rate. For the mated pair of Whooping Cranes modeled in this study, energy costs were estimated using a combination of time-activity budgets and energy costs of different behaviors for birds, obtained from the literature.

To obtain time budgets, each crane was videotaped during the study period, and behaviors were later sampled from videos. The cranes were videotaped as consistently as possible during all daylight hours between a blood draw on the morning of 9/24/12 (11:48 AM for the male, 11:41 AM for the female) and a second blood draw on the morning of 9/28/12 (10:58 AM for the male, 10:43 AM for the female). Videos were taken between the two blood draws because the doubly-labeled water technique produces a measurement of total energy expenditure specifically between these two draws. For reference, filming times and sunrise/sunset times are shown in S1 Table.

The behavior of each crane was sampled instantaneously every 30 seconds throughout the experiment period. For example, if filming began at 6:49 AM, the cranes behavior at 6:49:00, 6:49:30, 6:50:00, etc., was recorded. Behaviors were categorized to the categories shown in Table 1.

A basic assumption of time budget studies is that study subjects engage in the same types of behavior in the same relative frequencies while out of sight as they do while in sight. Thus, days and times of day that cranes were out of sight were considered in relation to daily behavior patterns. The proportions of time that cranes spent in various broad categories of behavior (comfort, foraging, walking, resting, social) were also examined overall, across days, and across different times of day in order to identify any patterns that might affect overall energy cost calculations.

The overall proportion of time that the cranes were out of sight was low (5.8% for the female and 12.9% for the male). Time out of sight was inflated by three periods of time for which videos were lost (due to computer errors while videos were copied). Missing videos account for 0.8% of the total time the female was observed and 7.6% of the time the male was observed. When missing videos are accounted for, the male and female were out of sight for approximately the same proportions of time (5.3% and 5.0%, respectively). The other primary causes of “out of sight” observations were delays in camera movement to follow the focal crane and periods of time when the cranes were in thick vegetation, on the opposite side of the berm, or on the west side of the pen where our view was blocked by the cranes’ indoor enclosure.

Overall, the male and female engaged in similar behavior patterns during the study period (S5 Fig.). The proportions of time that the female spent in various behaviors were fairly consistent across days, while the male showed more variation in behavior by day (S6 Fig.). S7 Fig. shows the proportions of time that each crane spent in various behaviors by hour of the day (i.e., hour 7 = 7:00-7:59). Time spent in different behaviors varied among hours of the day, but overarching patterns could not be discerned.

Because behavior patterns by time of day were difficult to discern, potential for bias caused by periods when the birds were out of sight was uncertain. Proportions of the day spent in each behavior were calculated in two ways. First (Method 1), overall proportions of time in sight spent in each behavior were calculated for each day using the equation:

$$P_{i}=\frac{N_{i,day}}{N_{tot,day}}$$

where P­_i_ is the proportion of the day spent in behavior i, N_i,day_ is the number of observations of behavior i for the day, and N_tot,day_ is the total number of in-sight behavior observations for the day. The male crane was out of sight for the entire period of hours 14 and 15 on 9/24 due to accidental loss of video from that period. To compensate for the missing hours, the average count of each behavior was calculated specifically for hours 14 and 15 for the other days of the study. These values were averaged. The average hour 14 and hour 15 values were added to N_i,day_ for 9/24, and N_tot,day_ was increased accordingly.

Second (Method 2), proportions of time spent in each behavior were calculated for each hour of each day, and the hourly proportions were averaged:

$$P_{i}=\frac{\sum_{h=1}^{H} \frac{N_{i,h}}{N_{tot,h}}}{H}$$

where N_i,h_ is the number of observations of behavior i in a given hour, N_tot,h_ is the total number of in-sight behavior observations for that hour, and h represents the hour (ranging from 1 to H, the total number of hours that the cranes were observed in a given day). To compensate for the missing hours 14 and 15 on 9/24, proportions of time spent in each behavior during hours 14 and 15 for the other days of the study were calculated and averaged. These average values were included in the daily average for 9/24, with H increased accordingly.

Final activity-energy budgets for daylight hours were calculated by finding the average proportion of time spent in each type of behavior for each day. Each behavior was assigned an energy cost from the literature (Table 1). Average daily energy expenditure on activity for each day (E_day_) was calculated as the average energy cost of activities, weighted by time spent in each activity, as shown in the following equation:

$${E_{day}=P}_{RS}*E_{RS}+P_{RA}*E_{RA}+P_{F}{*E}_{F}+P_{W}*E_{W}+P_{C}*E_{C}+P_{AC}*E_{AC}+P_{UC}*E_{UC}+P_{D}*E_{D}$$

where P represents proportion of daily time spent in a behavior and E represents energy cost of that behavior (as a multiple of basal metabolic rate). The subscripts abbreviate behaviors as follows: RS = rest-sleep, RA = rest-alert, F=forage, W=walk, C = comfort, AC = alarm call, UC = unison call, D = dance.

S2 Table shows the estimated energy expenditure on activity for each crane during the active period, calculated using these two methods. The method used to calculate time spent in different activities made little difference in the daily energy expenditure calculated. Differences between Method 2 and Method 1 ranged from -0.05 x basal metabolic rate (9/25 for the female) to 0.06 x basal metabolic rate (9/26 for the male), or from -51 kJ/day to 71 kJ/day. (See Table 2 for basal metabolic rates.)

Using both methods, estimated active period energy expenditure for the male and female were similar. This is likely because they spent similar amounts of time in the same activities, and because the energetic costs assigned to rest-alert, comfort behavior, and foraging were similar.
