# Supplementary material for: Validation of a Mechanistic Model for Non-Invasive Study of Ecological Energetics in an Endangered Wading Bird with Counter-Current Heat Exchange in its Legs
Source: PLoS One. 2015 Aug 26;10(8):e0136677. doi: 10.1371/journal.pone.0136677 (PMC4550283; doi:10.1371/journal.pone.0136677)
Supplement: S3 Text — (DOCX) [file pone.0136677.s016.docx]

To estimate temperatures experienced by wild migratory Whooping Cranes, monthly means of daily minimum and maximum air temperatures were downloaded from the NOAA National Climate Data Center website (http://www.ncdc.noaa.gov/oa/climate/ stationlocator.html). Data from all available years were downloaded from weather stations in Necedah, WI (1954-2008), Aransas National Wildlife Refuge (1941-2008), and Weeki Wachee, FL (near Chassahowitzka National Wildlife Refuge; 1970-2008). Air temperature data from Fort Smith, Northwest Territories (near WBNP; 1913-2008) were collected from Environment Canada’s National Climate Data and Information Archive (http://climate.weatheroffice.gc.ca/ climateData/canada_e.html). The 5th percentile of all daily minimum temperatures available and the 95th percentile of daily maximum temperatures were examined for each month that cranes would be in each location. The minimum and maximum temperatures were rounded to the nearest 5°C interval.
